# A Novel Regulatory System in Brain Development and Death Driven by a Bioactive Peptide Linked to a Specific Isoform of Acetylcholinesterase

**DOI:** 10.3390/biom16091336

**Published:** 2026-09-15

**Authors:** Adam Mohammed Khan, María-Salud García-Ayllón, Owen Hollings, Sara Garcia-Ratés, Susan Greenfield

**Affiliations:** 1 Neuro Bio Ltd., Building F5, Culham Science Centre, Abingdon OX14 3DB, UK; adam.khan@neuro-bio.com (A.M.K.); owenhollings@gmail.com (O.H.); susan.greenfield@neuro-bio.com (S.G.); 2 Unidad de Investigación, Hospital General Universitario de Elche, FISABIO, 03203 Elche, Spain; ms.garcia@umh.es; 3 Instituto de Neurociencias de Alicante, Universidad Miguel Hernández-CSIC, 03550 San Juan de Alicante, Spain; 4 Centro de Investigación Biomédica en Red sobre Enfermedades Neurodegenerativas (CIBERNED), 03550 San Juan de Alicante, Spain

**Keywords:** AChE-T AChE-R, T14, development, Alzheimer’s disease

## Abstract

Background: Acetylcholinesterase (AChE) demonstrates non-enzymatic actions attributable to a 14mer bioactive peptide derived from its sixth exon, ‘T14’, a pivotal agent driving Alzheimer’s disease (AD). Methods: To investigate this possible parallel between development and neurodegeneration, we tracked the expression and timeline of T14 in embryonic and neonatal rodent brain tissue in relation to the expression of its parent molecule, AChE, benchmarked against markers of neural maturation (NeuN) and neurodegenerative pathology (pTau) using sedimentation profiling, Western blotting, and qPCR. Results: We report two key findings: Firstly, while overall AChE activity, expression, and oligomeric assembly in the developing rodent cortex predominantly reflect AChE-T, endogenous T14 appear to selectively correspond with the AChE-R variant (r = 0.936, *p* = 0.0639), peaking at P7 before declining in tandem with NeuN and pTau. Secondly, application of exogenous peptide to cultured SHSY-5Y cells triggers the selective upregulation of this same variant, AChE-R (F_(3,18)_ = 9.320, *p* < 0.05). This effect is blocked by cotreatment with either mTORC1 inhibitor rapamycin or NBP14, an antagonist of T14. Conclusions: We conclude that in development, T14 and AChE-R are closely linked, that both are involved in a developmental mechanism most active in the embryonic brain, and that inappropriate reactivation of this mechanism in the mature brain could be the driver in the progression of AD.

## 1. Introduction

It has been known for many decades that acetylcholinesterase (AChE) can have alternative, non-enzymatic roles in both development [1,2] and neurodegeneration [3,4,5]. These actions have been attributed to a 14mer peptide, derived from the AChE C-terminus, ‘T14’ [6]. In neurodegeneration specifically, T14 has been shown elicit cell signalling selectively through allosteric modulation of α7-nAChRs and upstream of amyloid beta, reviewed in [6].

T14 can be produced through proteolytic cleavage of the C-terminus of the major AChE isoform ‘AChE-T’, encoded by exon 6 of the gene [7,8,9]. However, it is the minor ‘read-through’ isoform (AChE-R) [3,10] that is more dominant during rodent embryogenesis [11] and which increases in adult neurodegeneration [12]. This expression pattern of AChE-R corresponds to the developmental [13,14] and pathological [15,16] profiles of T14. Surprisingly, however, AChE-R protein does not appear to include the provenance of T14, exon 6 [9]: hence, the aim of this research was to explore this apparent paradox. Structurally, these two isoforms can be differentiated: AChE-T exists as a tetramer when PRIMA is incorporated, whereas AChE-R remains as a monomer [17].

First, we determined AChE variant expression and the oligomerisation pattern of AChE forms by real-time quantitative PCR (RT-qPCR) assays and gradient sedimentation assays in the rat brain and compared these profiles with that of T14 over the same period. Secondly, we compared the developmental profile of T14 in relation to AChE variants by benchmarking against the marker of neuronal maturation RNA Binding Fox-1 Homolog 3 (NeuN, [18]) and the expression patterns of AD marker phosphorylated tau (pTau, [19]).

To complement ontogenic data, we evaluated AChE splice variant expression by RT-qPCR in human neuroblastoma SH-SY5Y cells treated with T30, the more stable parent molecule of T14 [8]. Cells were treated with T30 alone or cotreated with T30 alongside either the cyclic T14 antagonist, NBP14, or rapamycin, an inhibitor of mTOR acting downstream of T14 [20].

## 2. Materials and Methods

### 2.1. Animals

These experiments were performed in accordance with the 1964 Declaration of Helsinki and its later amendments and with the appropriate approval of the Ethics and Scientific Committees (Miguel Hérnandez University, Code: 2016/VSC/PEA/00066).

Embryonic day 14, 16, 18, and 20, and postnatal day 5, 7, 14, 21, 25, 35, and 60 Wistar rats were used. After the postnatal P7 stage only male rats were employed. Parental rats were obtained from Charles River (Margate, UK). Rats were immediately culled using an appropriate Schedule 1 method in accordance with the UK Animals (Scientific Procedures) Act 1986. Hemicortices were dissected and stored at −80 °C except for E14 brains, for which the entire telencephalon was dissected.

Postnatal day 7, 14, 21 and 60 mice were obtained from Charles River (Harlow, UK). Mice were selected from litters without regard to sex, and sex was not recorded because reliable determination at earlier postnatal stages was not routinely performed. Therefore, the study likely included both male and female mice, although sex distribution cannot be confirmed. Mice were culled using an appropriate Schedule 1 method in accordance with the UK Animals (Scientific Procedures) Act 1986. Brain tissue was snap frozen and stored at −80 °C. Whole P7, P14 and P21 mouse brains were used, while brains from P60 mice were cut between the hemispheres and one hemisphere used.

### 2.2. Homogenisation of Tissues and Solubilisation of Proteins

Rat brain tissue was homogenised in 10% *w*/*v* ice cold tissue lysis buffer (Tris-HCL 50 mM, NaCL 500 mM, EDTA 5 mM, Nonidet P-40 1%, Triton X-100 0.5% (all from Thermo Fisher Scientific, Swindon, UK), and Roche (Basel, Switzerland) cOmplete™, Mini, EDTA-free Protease Inhibitor Cocktail tablet 1: 25 mL) with 20–22 strokes of a Potter-Elvehjem glass homogeniser. Samples were then sonicated 3× in 15 s bursts using a Decon FS-100 (Decon Laboratories Limited, Hove, UK) and centrifuged at 16,000× *g* at 4 °C and the supernatant aspirated and stored at −80 °C until required.

Mouse brain tissue was homogenised using pestle and Dounce homogenisers in 33% *w*/*v* of lysis buffer containing protease inhibitor (cOmplete™ ULTRA Tablets, Mini, EDTA-free, EASYpack Protease Inhibitor Cocktail, Roche, Basel, Switzerland) and phosphatase inhibitor (PhosSTOP, Roche, Basel, Switzerland) cocktails dissolved in 10 mL 1× PBS (ThermoFisher Scientific, Swindon, UK). Samples were centrifuged at 12,470× *g* at 4 °C for 30 min and the supernatant aspirated and stored at −80 °C until required.

Protein content of supernatants was quantified using the Thermo Scientific Pierce 660 nm Protein Assay according to the manufacturer’s instructions with a CLARIOstar Plus plate reader (BMG Labtech, Aylesbury, UK).

### 2.3. Enzymatic Activity and Total Protein Determination

AChE activity in protein extracts was determined by a modified microassay version of the colorimetric Ellman’s method [21] using 1 mM acetylthiocholine as substrate and 50 µM tetraisopropyl pyrophosphoramide (Iso OMPA) (Merck, Gillingham, UK) to inhibit butyrylcholinesterase, cholinesterase that co-exists with AChE. One milliunit (mU) of AChE activity was defined as the number of nmoles of acetylthiocholine hydrolysed per min at 22 °C.

To normalise AChE activity, protein concentrations of the samples were determined using the bicinchoninic acid method, with bovine serum albumin as standard (Pierce, Rockford, IL, USA).

### 2.4. Sedimentation Analysis

Molecular forms of AChE were separated according to their sedimentation coefficients by ultracentrifugation on 5–20% (*w*/*v*) sucrose gradients containing 0.5% (*w*/*v*) Triton X-100 [21]. Ultracentrifugation was performed at 250,000× *g* in a TLS 55 Beckman rotor for 4 h, at 4 °C. After centrifugation, 30 fractions were collected from the top of the tube and assayed for AChE activity to identify individual AChE forms (G4 = tetramers; G1 = monomers) by comparison with the position of molecular weight markers, catalase (11.4 S) and alkaline phosphatase (6.1 S). We estimated the AChE activity of G4 and G1 peaks calculating the sum of absolute activity of the fractions of the peak.

### 2.5. Western Blot

Western blot analysis was performed as previously described [13], with minor modifications. T14 expression was assayed in cortical and subcortical rat brain samples. Protein extract (80 μg protein per well) was mixed with Pierce™ LDS Sample Buffer (Non-Reducing, 4×) (ThermoFisher Scientific, Swindon, UK) and 10X Bolt™ Sample Reducing Agent (50 mM DTT final concentration) (ThermoFisher Scientific, Swindon, UK). Samples were heated at 60 °C for 10 min and then stored at −80 °C until use. Protein extracts (15 µg protein per well) were mixed with loading buffer (Bio Rad, Cat # 161-0747, Hercules, CA, USA) and β-mercaptoethanol (Bio Rad, Cat # 161-0710, Hercules, CA, USA). Samples were heated at 95 °C for 5 min and then stored at −80 °C until use. Rat proteins were separated on Bolt™ 4–12% Bis-Tris Mini Protein Gels, whilst mouse proteins were separated on 4–20% precast polyacrylamide gels (Bio Rad, Cat # 456-1094, Hercules, CA, USA) by electrophoresis. Then proteins were blotted on polyvinylidene fluoride membranes (PVDF; 0.2 μm) (Immobilon-P, Sigma-Aldrich, Cat # P2938, Darmstadt, Germany) and stained to evaluate the transfer quality with Ponceau red staining [22].

Ponceau signal was imaged using a CCD camera (G-Box, Syngene, Cambridge, UK) to determine the total protein content. Membranes were subsequently blocked for 1 h at room temperature using 5% non-fat dried milk in Tris-buffered saline with 0.05% Tween 20 (TBS-T) before overnight incubation at 4 °C with primary antibodies as follows: rabbit polyclonal T14 (1:1000, custom made by Genosphere, Paris, France), rabbit polyclonal NeuN (1:5000, Abcam, ab104225, Cambridge, UK), mouse monoclonal pTau Ser202/Thr205 (1:1000, Thermo Fisher Scientific, MN1020) (ThermoFisher Scientific, Swindon, UK). The specificity of T14 antibody is described by [7]. For T14 immunodetection, immunoneutralisation was performed by preincubating T14 polyclonal antibody diluted 1:1000 in TBS-T plus 5% dried skimmed milk, with T14 at 1 mg/mL for 3 h prior to application to the PVDF membrane following transfer. Antibody binding was resolved by incubation for 1 h with respective HRP-conjugated secondary antibodies: goat anti-rabbit IgG (1:10,000; Invitrogen, Swindon, UK) and goat anti-mouse IgG (1:10,000 Thermo Fisher Scientific, Swindon, UK). Immunoreactive signal was visualised with enhanced chemiluminescence-based detection (Bio-Rad, Watford, UK, Clarity Western ECL Substrate, #1705061) and a CCD Camera (G-Box, Syngene, Cambridge, UK) gel system. Scanned blots were visualised using GeneSnap software 7.1 (Syngene, Cambridge, UK). Protein levels were determined using ImageJ software 1.53 (NIH, Bethesda, MD, USA). Signal intensity was quantified by measuring optical density (O.D.) of the band of interest and normalising to that of the Ponceau loading control for mouse samples. Mouse T14 and pTau O.D. measurements were normalised to a P14 sample present on each membrane, whilst Rat T14 O.D. measurements were normalised to E14 for cortical samples and E16 for subcortical samples. Normalised optical density measurements were scaled within each dataset to facilitate comparison.

### 2.6. SH-SY5Y Cell Culture

SH-SY5Y human neuroblastoma cells were cultured in DMEM/F12+GlutaMAX™-I (Dulbecco’s modified Eagle medium; GIBCO Invitrogen Corporation, Swindon, UK) supplemented with 10% heat-inactivated foetal bovine serum (FBS), penicillin (100 U/mL), and streptomycin (100 μg/mL) and maintained at 37 °C in saturated humidity containing 95% air and 5% CO_2_. Cells were seeded at a density of 8 × 105 cells on 35 mm tissue culture dishes and after 24 h, treatment with 0.7 µM T30 (Genosphere) in DMEM/F12+GlutaMAX™ with 0.1% FBS was added. Cotreatment conditions utilised 0.5 µM NBP14 (Genosphere), 1 µM rapamycin (Sigma-Aldrich). After 24 h or 48 h cells were washed with Phosphate-saline buffer, suspended in 800 µL TRIzol reagent (Invitrogen^TM^, Carlsbad, CA, USA) and stored at −80 °C until RNA isolation. T30 and NBP14 were synthesised by Genosphere Biotechnologies (Paris, France).

### 2.7. RNA Isolation and Analysis of Transcripts by RT-qPCR

Levels of the transcripts that encoded AChE-T and AChE-R were analysed in rat brain and SH-SY5Y cells by RT-qPCR. Total RNA was extracted using the TRIzol reagent (Invitrogen^TM^, Carlsbad, CA, USA) in the PureLink^TM^ Micro-to-Midi Total RNA Purification System (Invitrogen^TM^ Life Technologies, Foster City, CA, USA) following the manufacturer’s instructions. First-strand cDNAs were synthesised by reverse transcription of 1.5 µg of total RNA using the High-Capacity cDNA Reverse Transcription Kit (Life Technologies, Thermo Fisher Scientific, Swindon, UK), according to the manufacturer’s instructions. Real-time quantitative PCR (RT-qPCR) amplification was performed on a QuantStudio3 (Applied Biosystems, ThermoFisher Scientific, Rockford, IL, USA). For determination of AChE transcript levels, Power SYBR^®^ Green PCR Master Mix (Applied Biosystems, Carlsbad, CA, USA) was used according to the manufacturer’s instructions and the corresponding primers. For rat brain samples the primers employed were: AChE-T forward 5′-CAGCAATACGTGAGCCTGAA-3′, reverse 5′-CTCGTCCAGCGTGTCTGT-3′, AChE-R forward 5′-CTCAGCGCCACAGGTAGG-3′, reverse 5′-TCTCTCCCGTCCTTCCAAC-3′; GAPDH forward 5′-TGGGAAGCTGGTCATCAAC-3′, reverse 5′-GCATCACCCCATTTGATGT-3′.

For SH-SY5Y cells the primers used were: AChE-T forward 5′-CTTCCTCCCCAAATTGCTC-3′, reverse 5′-TCCTGCTTGCTGTAGTGGTC-3′; AChE-R forward 5′-CTTCCTCCCCAAATTGCTC-3′, reverse: 5′-GGGGAGAAGAGAGGGGTTAC-3′; GAPDH forward 5′-AGCCACATCGCTCAGACAC-3′, reverse 5′-GCCCAATACGACCAAATCC-3′. PRiMA-1 transcript quantification was done with the TaqMan GenExpression Assay (Hs00930435_m1 for PRiMA-1 and Hs03929097 for GAPDH; Life Technologies) using TaqMan PCR Master Mix (Life Technologies). For each transcript, efficiency of the qPCR assay was assayed performing a relative standard curve using brain or SH-SY5Y control samples. Since resulting efficiency of the reactions was similar (between 89 and 91%), transcript levels were calculated by the comparative 2^−ΔCt^ method with respect to GAPDH.

### 2.8. Statistical Analysis

Statistical analysis was performed using GraphPad Prism 10 (v10.1.2; GraphPad Software Inc., La Jolla, CA, USA). Data were determined to be parametric using the D’Agostino–Pearson test. Simple linear regression was performed and Pearson’s correlation coefficient (r) was determined to assess linear correlations between variables. To analyse differences between the early and later stages of rat and mouse brain development, one-way analysis of variance (ANOVA) followed by Tukey’s multiple comparisons post hoc test was used to determine whether the means of three or more data groups differed significantly. For the SH-SY5Y qPCR data, one-way ANOVA followed by Fisher’s LSD post hoc test was used to compare each treatment group to its respective vehicle control, and outliers were designated by the modified Thompson Tau method, resulting in the removal of one datapoint from 8 of the 16 groups. Significant differences are indicated with an asterisk (*). *p* < 0.05 was considered significant throughout; data are expressed as the mean ± standard error of the mean (SEM).

## 3. Results

### 3.1. AChE-T Expression and Oligomerisation in the Rodent Cortex

**Figure 1:**
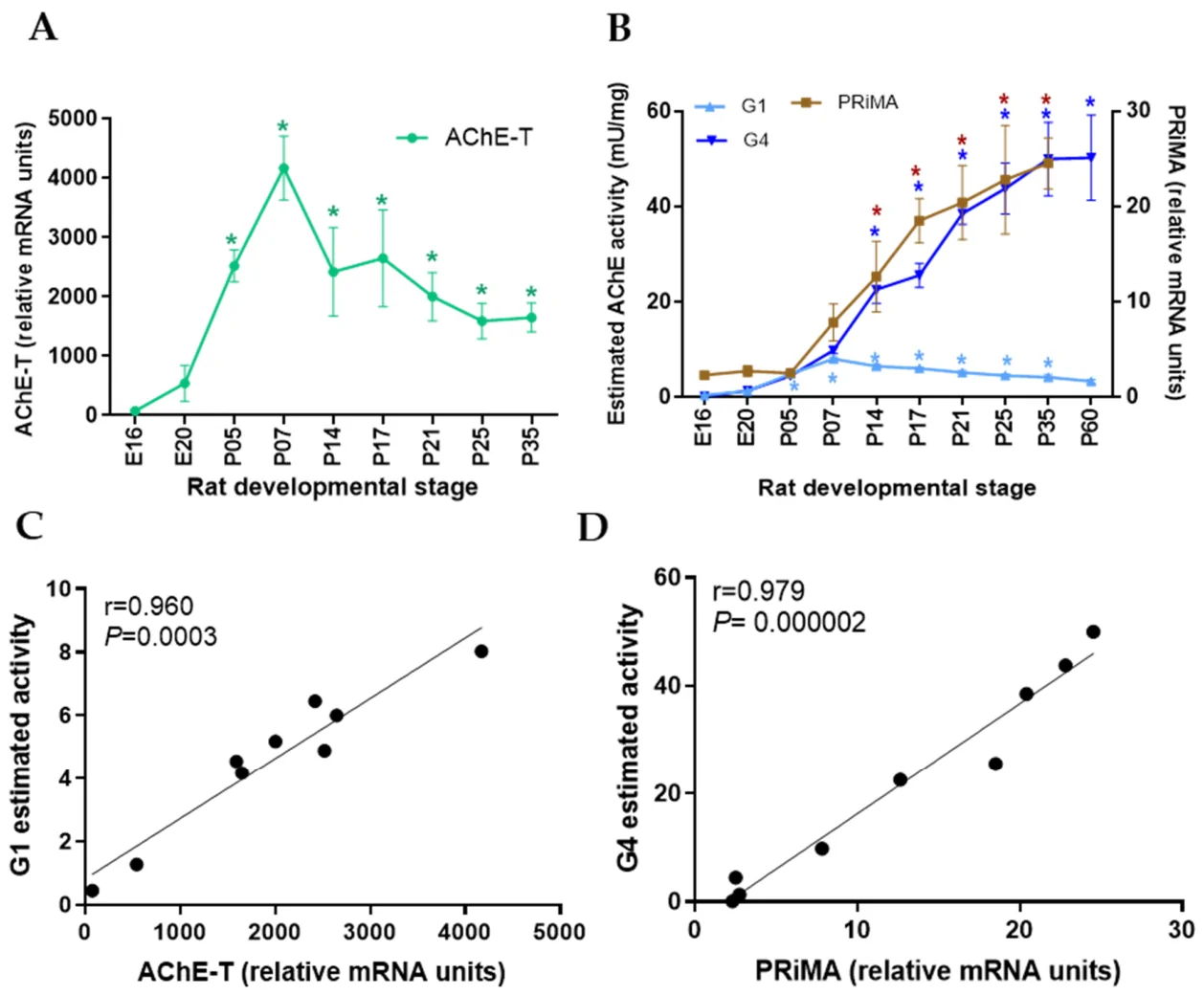
AChE-T in the developing rodent cortex. (**A**) Relative mRNA levels of the major AChE splice variant present in the brain, AChE-T, were analysed using qPCR in the developing rat brain cortex at embryo and postnatal stages. (**B**) Cortical levels of monomeric (G1) and tetrameric (G4) were determined by sedimentation profiling and AChE activity assay, left axis. PRiMA-1 transcript levels were determined by RT-qPCR and plotted, right axis. (**C**) Correlation between AChE-T mRNA and estimated G1 activity. (**D**) Correlation between levels of PRiMA mRNA and estimated G4 activity. For (**C**,**D**), *p* and r values from the Pearson test are included in the graph. N = 3–7 rat cortices. Statistical significance was determined by one-way ANOVA followed by Tukey’s post hoc test. Significant differences (*p* < 0.05) relative to E16 (**A**,**B**) are indicated (*).

AChE-T mRNA expression in rat cortical extracts was highest postnatally, peaking at P7 compared with baseline levels at E16 (F_(8,32)_ = 27.57, *p* < 0.05) and declining steadily thereafter (Figure 1A). Abundance in the same cortex samples of the mRNA that encoded PRiMA-1, the subunit anchor of the AChE-T tetrameric forms to the membrane, were in a low steady state but increased from P7 onwards (Figure 1B), being significant (F_(8,32)_ = 12.61, *p* < 0.05) from the P14 stages. After sedimentation analysis we estimated that enzymatic activity and activity of the monomer G1 increased significantly (F_(10,51)_ = 54.85, *p* < 0.05) until this time point and declined thereafter (Figure 1B), whereas G4 AChE steadily increased throughout development, before levelling out at P60, with levels approximately 6-fold higher than G1. AChE-T mRNA and G1 AChE estimated activity correlated (r = 0.960, *p* = 0.00003; Figure 1C), as did levels of PRiMA mRNA and estimated G4 AChE activity (r = 0.979, *p* = 0.000002; Figure 1D).

### 3.2. Cortical T14 Expression Corresponds More to AChE-R, than to AChE-T

**Figure 2:**
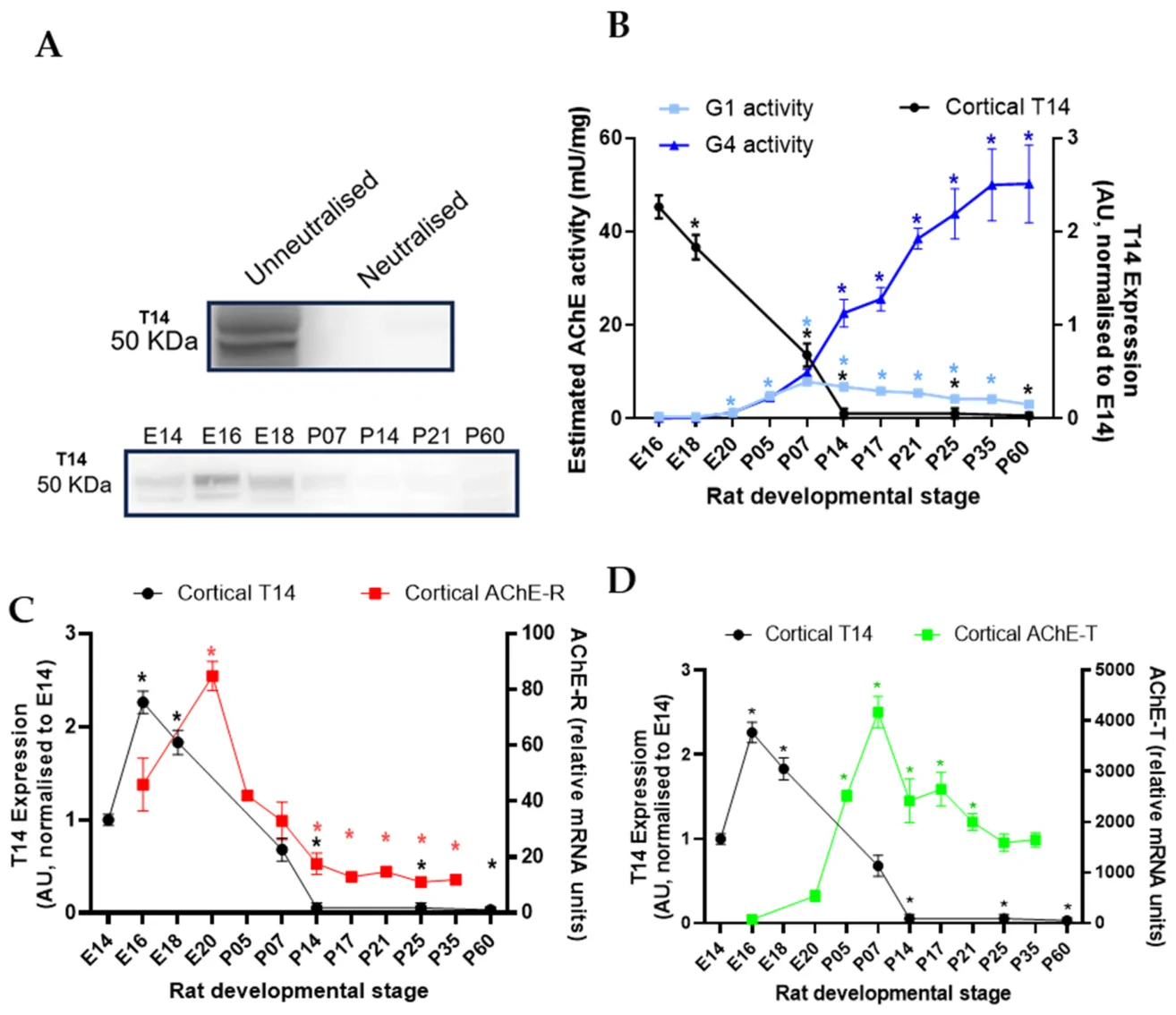
Cortical T14 expression corresponds with AChE-R, not AChE-T. (**A**) Western blot immunoneutralisation of T14 antibody by incubation with T14 1000× in excess of the antibody prior to incubation with the membrane. Representative blot of cortical T14 immunoreactivity. (**B**) Quantification T14 levels determined by Western blot in the cortex, normalised to E16, right axis, plotted against estimated cortical G1 and G4 AChE activity, right axis. (**C**) T14 in the cortex determined by Western blot, left axis, plotted against estimated cortical AChE-R mRNA, right axis. (**D**) T14 in the cortex, left axis, plotted against estimated cortical AChE-T mRNA, right axis. T14 data presented in (**B**–**D**) are from the same experiment, and AChE-T mRNA levels reported in (**D**) are also reported in Figure 1A. (**A**–**D**) N = 3–7 rat cortices. Statistical significance was determined by one-way ANOVA followed by Tukey’s post hoc test. Significant differences (*p* < 0.05) relative to E16 (AChE—(**B**–**D**); cortical T14—(**B**)), or E14 (cortical T14—(**C**,**D**)) are indicated (*). The original western blot images can be found at Figure S1.

We analysed expression levels of T14 across rat brain development. The results from Western blotting showed a double immunoreactive band at approximately 50 kDa, completely blocked by immunoneutralisation (Figure 2A). T14 immunoreactive levels were plotted with G1 and G4 AChE levels, and the results showed that following P7, where G4 activity dominates over G1 (*p* < 0.05 with respect to E16), Figure 2B), cortical T14 expression decreases and remains low/non-existent across P14–P60 (F_(5,13)_ = 95.46, *p* < 0.05 with respect to E16). Whilst the peak in G1 activity seen at P7 appears to correspond to the highest cortical expression of T14 measured in postnatal brain tissue (Figure 2B), T14 levels in embryonic brain tissue were higher overall and maximal at E16, before rapidly diminishing and tending towards zero postnatally (Figure 2). At embryonic stages, levels of AChE-R transcript were most elevated, peaking at E20 (F_(8,32)_ = 39.96 *p* < 0.05 with respect to E16) and declining postnatally (Figure 2C), whereas AChE-T transcript peaks at postnatally at P7 (F_(8,32)_ = 27.57, *p* < 0.05 with respect to E16; Figure 2D). Hence, correlation assays demonstrate that the pattern of T14 expression corresponded more strongly with AChE-R (r = 0.936; *p* = 0.0639) than AChE-T (r = −0.576; *p* = 0.4242), despite the inevitable lack of precision using only four timepoints that matched.

### 3.3. T14, NeuN and pTau Show Comparable Expression Profiles in Development

**Figure 3:**
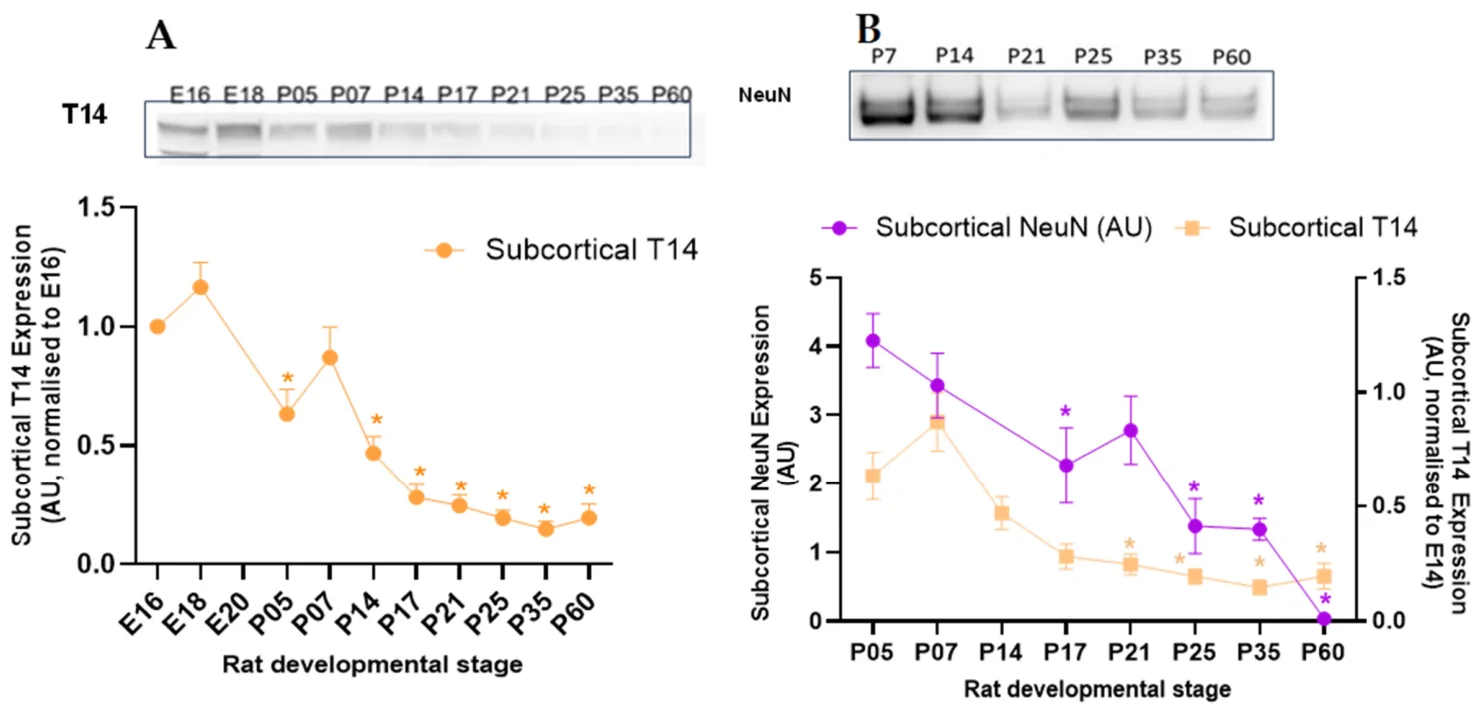

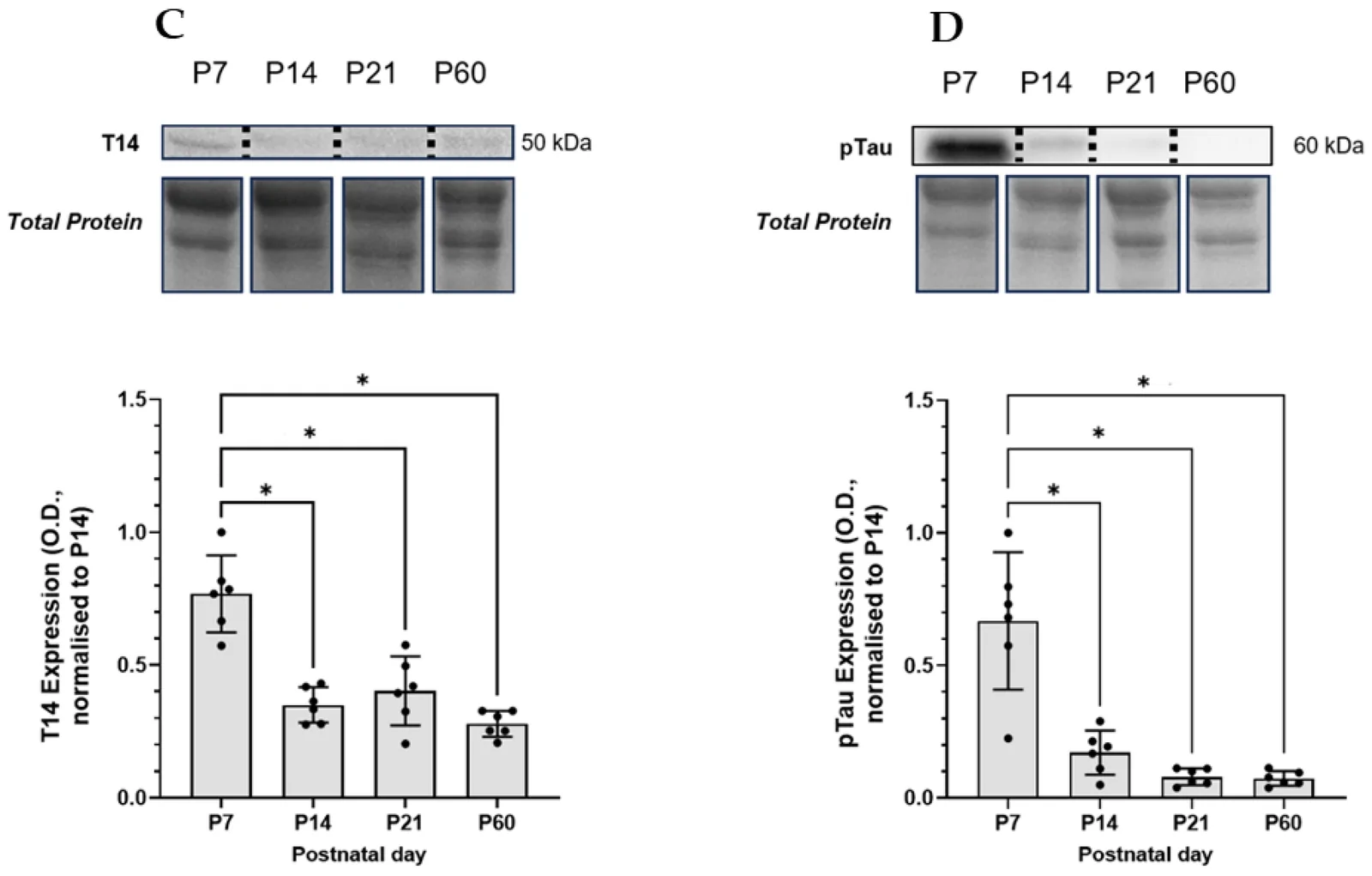
T14, NeuN, and pTau show comparable expression profiles during postnatal rodent brain development. (**A**) Representative immunoblot of T14 immunoreactivity (50 KDa) in rat subcortex; expression plotted across age. (**B**) Representative immunoblots of NeuN immunoreactivity (49 KDa) in postnatal subcortical rat brain; expression plotted across age. Subcortical expression of T14 is also plotted, right axis. (**C**) Representative immunoblots of T14 immunoreactivity and total protein stain, bar graph showing T14 expression in whole mouse brain across age. (**D**) Representative immunoblots of pTau (Ser202, Thr205) immunoreactivity and total protein stain, bar graph showing pTau expression in whole mouse brain across age. T14 data presented in (**A**,**B**) are from the same experiment. (**C**,**D**) show data normalised to total protein content and a control P14 sample present on each membrane (arbitrary units, AU). (**A**,**B**) N = 3–7 per age. (**C**,**D**) Dots represent individual mice. N = 5–6 per age, a total of 23. Statistical significance was determined by one-way ANOVA followed by Tukey’s post hoc test. Significant differences (*p* < 0.05) relative to E16 (subcortical T14—(**A**); cortical T14—(**B**)), P05 (subcortical T14—(**B**); subcortical NeuN, (**B**)), and P7 (T14—(**C**); pTau—(**D**)) are indicated (*). The original western blot images can be found at Figure S2.

In the subcortical brain T14 expression declines with a distinct peak in observed at P7 (Figure 3A). The following experiments showed that in rat subcortical samples NeuN expression decreases in postnatal stages in the rat subcortex, corresponding to levels of T14 across ageing, i.e., they both decrease after P7 steadily (Figure 3B). T14 in whole mice brains showed similar expression patterns to rat, namely, a significant age-dependent decrease after the P7 stage in postnatal brain tissue (F_(3,20)_ = 25.50, *p* < 0.05; Figure 3C). Levels of the phosphorylated tau protein (pTau) were also measured in mice brains by Western blot, showing a similar significant age-dependent decrease when compared to P7 (F_(3,20)_ = 25.39, *p* < 0.05; Figure 3D) and parallel with T14 and NeuN.

### 3.4. Exogenous T30 Induces AChE-R Expression in SHSY-5Y Cells

**Figure 4:**
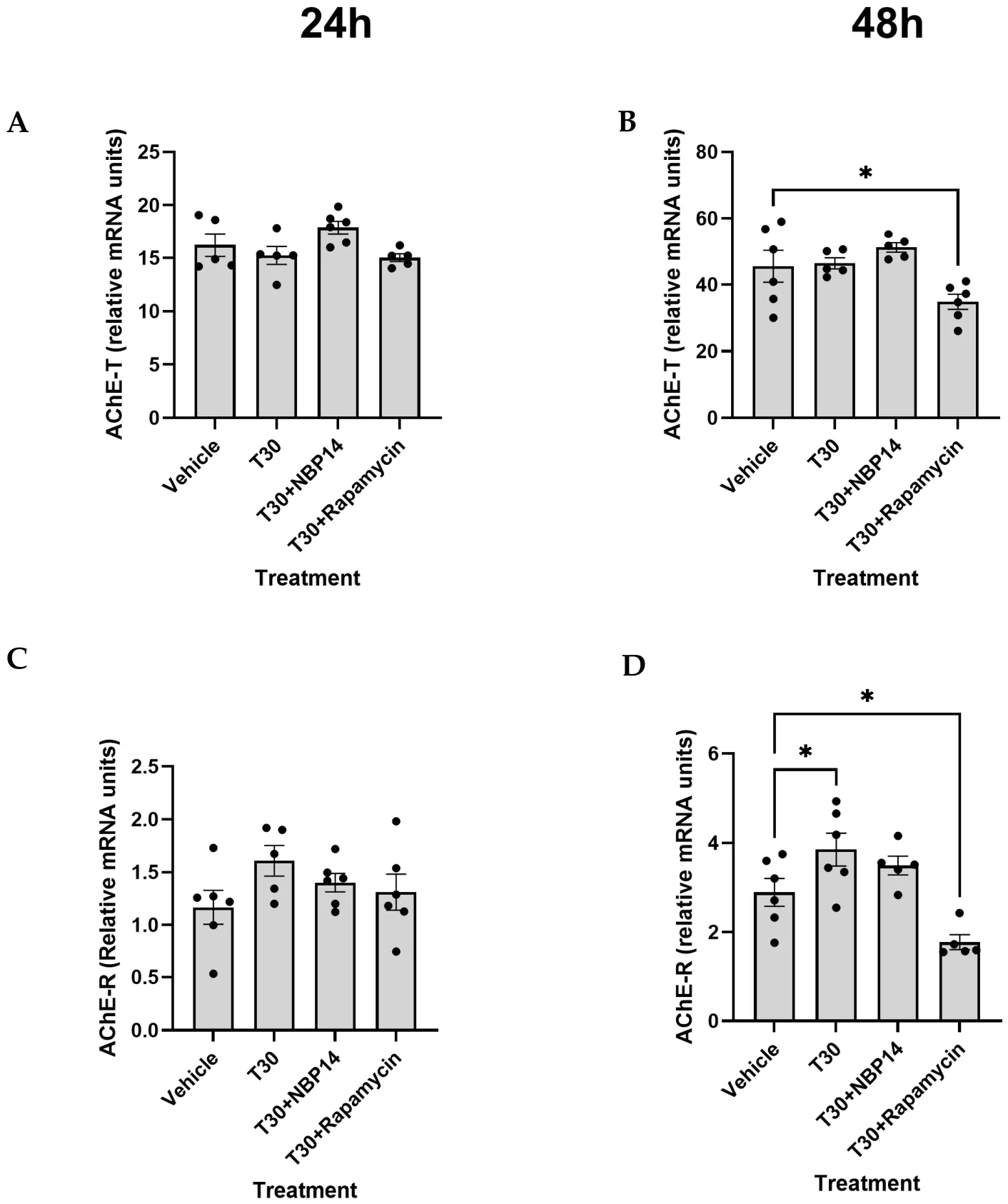
T30 increases mRNA expression of AChE-R, not AChE-T, 48 h after treatment. (**A**) Relative AChE-T mRNA expression, normalised to GAPDH mRNA, 24 h after treatment with T30, T30+NBP14, and T30+Rapamycin. (**B**) Relative AChE-T mRNA expression, normalised to GAPDH mRNA, 48 h after treatment with T30, T30+NBP14, and T30+Rapamycin. (**C**) Relative AChE-R mRNA expression, normalised to GAPDH mRNA, 24 h after treatment with T30, T30+NBP14, and T30+Rapamycin. (**D**) Relative AChE-R mRNA expression, normalised to GAPDH mRNA, 48 h after treatment with T30, T30+NBP14, and T30+Rapamycin. Dots represent biological replicates, where N = 5–6. Statistical significance was determined by one-way ANOVA followed by Fisher’s LSD post hoc test. Significant differences (*p* < 0.05) relative to vehicle are indicated (*).

To demonstrate the direct relation between T30, the more stable parent molecule of T14, and AChE, the SH-SY5Y cells were treated with T30 (0.7 µM). The results showed statistically significant difference amongst the mean AChE-T mRNA expression after 24 h treatment (F_(3,17)_ = 3.209, *p* < 0.05; Figure 4A) but no significant difference between the treatment groups based post hoc testing. AChE-R mRNA expression did not differ significantly after 24 h treatment (F_(3,19)_ = 1.526, n.s.; Figure 4C). However, at 48 h, a significant difference in AChE-R mRNA expression was observed (F_(3,18)_ = 9.320, *p* < 0.05; Figure 4D). Post hoc testing revealed a statistically significant increase in AChE-R mRNA expression, following T30 (0.7 µM) treatment (*M* = 3.85), in turn sensitive to NBP14 (0.5 µM) cotreatment (a cyclic antagonist of T14) (*M* = 3.49) and rapamycin (1 µM) cotreatment (downstream of T30) (*M* = 1.78), which reduced to levels below baseline/control (*M* = 2.89).

The same was not true for AChE-T, where whilst a significant difference in mRNA expression was observed after 48 h treatment (F_(3,18)_ = 5.101, *p* < 0.05; Figure 4B), post hoc testing revealed that only rapamycin cotreatment (*M* = 34.81) had significantly lower AChE-T mRNA expression than control (*M* = 45.50), with a mean difference of 10.69 (*SE* = 4.18, *p* < 0.05).

## 4. Discussion

Non-enzymatic functions of AChE have been implicated in both development [1,2] and neurodegeneration [3,4,5]. Here we draw a parallel between the potential role for AChE in both development and neurodegeneration through a common mechanism based on peptides derived from its C-terminus, namely, T14 and its immediate precursor T30. In the developing rodent brain, T14 and AChE-R exhibit a distinct co-expression profile that parallels changes in markers of both neuronal maturity (NeuN) and neurodegeneration (pTau) in the postnatal brain. Furthermore, we demonstrate that T30 selectively upregulates AChE-R mRNA expression in vitro.

The study was performed in brain samples from both rats and mice, suggesting the findings could be generalised across the rodent species. T14 expression was measured using Western blot, using a custom-made antibody, for which the epitope specificity has been characterised; the antibody selectively recognises the exposed T14 epitope without cross-reacting with full-length AChE or the extended T30 peptide [7]. We further confirm the specificity of T14 detection here using an immunoneutralised antibody control, which abolished signal. The primary immunoreactive signal detected at ~50 kDa is consistent with a stable T14-containing complex or precursor species. In Western blot homogenates, this band reflects T14-immunoreactive species rather than freely dissociated monomeric T14 (1.5 kDa), a distinction that has been maintained throughout our quantification and functional interpretation.

### 4.1. AChE-T Expression and Activity in the Neonatal Rodent Brain

Consistent with previous data that showed AChE expression and maximal activity around P7 [23,24,25], we observed a peak in AChE-T mRNA expression at P7 which declined thereafter. AChE-T is present as monomers, which can dimerise via disulphide bridging of cysteine residues at the C-terminus and oligomerise into homotetramers via further hydrophobic interactions with two additional monomers, presenting in the G4 form [3]. In the brain, G4 AChE-T is attached to the membrane by a subunit, proline-rich membrane anchor, ‘PRiMA’, which contains a proline rich attachment domain [17,26]. Anchoring via PRiMA sequesters the AChE C-terminus within the membrane, preventing degradation and promoting oligomerisation [27]. Accordingly, here we show that PRiMA expression increased sharply after P5, coinciding with a shift in oligomerisation of AChE towards the G4 form once it could be anchored in the membrane; hence the strong correlation seen here of G4 AChE with PRiMA levels is exactly what would be predicted.

The days around P7 are an extremely active period of growth and development in the rodent brain [28]. At this point we observed the highest AChE-T transcript levels, although enzymatic AChE activity was lower than in later stages. This mismatch supports the notion of a non-enzymatic role for AChE: it is known that AChE mRNA levels need not correlate with enzymatic activity [24], and indeed a pool of catalytically inactive AChE has been previously described in brain [29]. Hence, it is likely that AChE expression is elevated to this extent in the developing brain in a capacity outside of its traditional role [3,30].

### 4.2. T14 Expression During Development of the Rodent Brain

It seemed most likely that T14 levels would closely track AChE-T expression across age as only AChE-T includes the requisite Exon 6 that encodes the peptide sequence [31]. For the postnatal data, this indeed appeared to be the case; at P7, where AChE-T expression is increased but presenting as G1 (likely due to lower PRiMA expression), elevated T14 levels occurred in contrast to later stages, where an increase in G4 levels after P7 and a decline in T14 levels were seen.

However, the embryonic data revealed a paradoxically different pattern of T14 and AChE isoform expression. T14 levels were higher in embryonic tissue than in postnatal tissue, despite lower G1 levels. Instead, T14 levels correspond much more closely with AChE-R mRNA expression during the embryonic stages, and this pattern of correspondence can be seen across ontogeny. Only four datapoints were available, thus preventing an attempt at any formal correlation. However, the apparent near correlation between AChE-R and T14 expression would suggest that T14 is primarily derived from AChE-R during early development rather than AChE-T. In a pathological context, however, the two sources (AChE-R and AChE-T) may not be mutually exclusive.

### 4.3. T14, NeuN, and pTau Expression Indicate a Developmental Transition at P7

The decline in postnatal T14 expression after P7 parallels the postnatal NeuN and pTau expression seen, suggesting a transition from a highly plastic and proliferative state to a more mature neural environment. During early development, upregulated yet controlled phosphorylation of tau facilitates microtubule depolymerisation required for neurite outgrowth, mitotic spindle formation, and intracellular transport [32]. Here we detect the AT8 epitope (ser202, thr205)—strongly associated with Braak staging in AD [19]. Phosphorylation at this epitope is significantly higher at P7, more than later days, suggesting that, in normal development, phosphorylation of this epitope, implicated in AD pathology, is part of a physiological process that becomes inappropriately recapitulated in disease.

T14 acts as a biphasic positive allosteric modulator at α7 nicotinic acetylcholine receptors (α7-nAChRs) and, via activation of GSK-3 and mTORC1, can drive tau phosphorylation at the AT8 epitope as a possible early driver of Alzheimer’s disease pathology [15,20]. Our data showing a similar pattern of decline in T14 expression after P7 suggest that the same signalling cascade active in AD may also constitute a physiological process in the developing brain. The present findings point towards a process that occurs in the highly plastic, early developing brain in which tau is phosphorylated at the AT8 epitope. Yet, in the adult brain, that same phosphorylation event is associated with toxicity and neurodegeneration, suggesting that pathological processes in neurodegeneration recapitulate developmental signalling cascades [19].

As the brain matures beyond P7, expression of NeuN also declines in the subcortex, corresponding to the decline in subcortical T14 levels (Figure 3C). NeuN is a marker of neural maturity, and its decline could reflect the conclusion of major proliferative events and the onset of synaptic pruning, a key developmental process in the maturing brain [18]. Such developmental remodelling mirrors mechanisms of synaptic vulnerability seen in early neurodegeneration, where scaffold proteins like PSD-95 govern structural integrity and plasticity prior to classic histopathology [33]. Taken together, the expression patterns of T14, pTau, and NeuN point towards highly active metabolic events occurring at P7, which decline thereafter. P7 represents a well-characterised developmental window in the rodent brain [28]. While it remains unclear whether these pathways directly incorporate NeuN, they collectively suggest that metabolic events occurring at P7 involve molecules otherwise associated with AD pathology. It is possible that developmental regulation of AChE-R drives this process through the production of T14 and subsequent calcium influx via α7-nAChRs and that this same cascade of events facilitating growth and plasticity in development becomes inappropriately active in the aged brain, resulting in pathology [6].

### 4.4. T14 Drives a Feedforward Mechanism Through AChE-R Upregulation

T30 is the parent molecule of T14, and has been used previously in various preparations and in some cases more pronounced effects than T14, demonstrating capacity for α7-nAChR at an allosteric site [8]. In SH-SY5Y cells, after 48 h of treatment with T30 (0.7 µM), expression of AChE-R alone was selectively elevated, an effect not observed at 24 h. Cotreatment with NBP14 (0.5 µM) (an antagonist of T14) [6] inhibited this transcriptional effect, and rapamycin (1 µM), an inhibitor of mTORC1 which acts downstream of the receptor [20], reversed the effect to below control, indicating some basal tone in the absence of exogenous treatment. Since a comparable selective increase in AChE-R occurs in AD [12], this observation provides further evidence for the feedforward mechanism of T14/T30 proposed to occur in AD, now through apparent upregulation of the AChE-R isoform, in a time-dependent manner. Previous studies have shown that T14 increases the production of its target [34], potentially creating a positive feedback loop. Our findings provide further evidence for this loop, demonstrating isoform-specific (AChE-R rather than AChE-T) upregulation after treatment with T30. We cannot, however, exclude the possibility that AChE-T also contributes to the production of T14 in contexts such as neurodegeneration, especially considering that both degradation of G4 AChE and increased expression of G1 are associated with synaptic degeneration in AD [35].

## 5. Conclusions

The data presented here provide new insight into the provenance of T14 and its role in both physiological and pathological contexts. The parallel expression patterns of T14, pTau at the AT8 epitope, and NeuN at P7 demonstrate that molecules implicated in Alzheimer’s disease pathology play essential physiological roles during critical developmental windows. T14 appears to arise from different AChE isoform sources depending on developmental stage and context. During early embryonic development, T14 corresponds most strongly with AChE-R expression, while postnatal patterns may suggest contributions from AChE-T oligomeric breakdown. A limitation of these data is the reliance on discrete landmark developmental stages rather than a continuous daily embryonic series. While the current data show expression differences across major embryonic and early postnatal windows, higher-density sampling will be valuable in future work to pinpoint the precise chronological onset of changes in protein expression.

**Figure 5:**
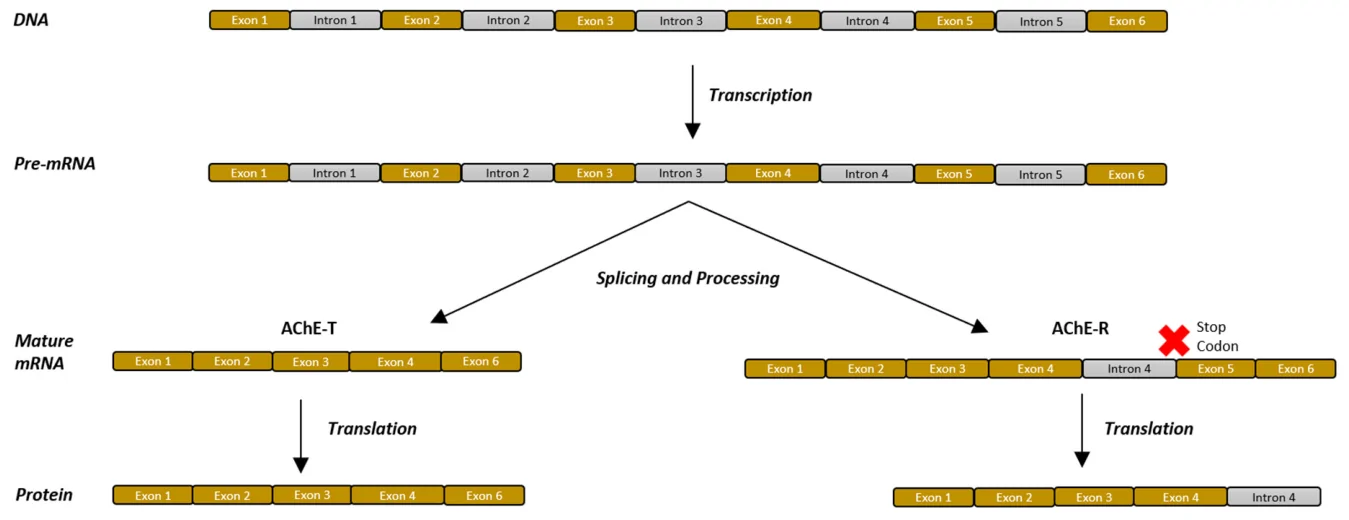
Diagram illustrating splice patterns of AChE pre-mRNA and the presence of exon 6 in each splice variant.

The correspondence between T14 and AChE-R expression presents an apparent paradox, since the mature AChE-R protein lacks exon 6 and therefore cannot contain the T14 sequence [9], illustrated in Figure 5. However, alternative possibilities could involve dysfunctional regulation and skipping of this stop codon or translation from an alternative open reading frame within the AChE-R mRNA [36]. The presence of exon 6 downstream of a stop codon in intron 4 within AChE-R mRNA could allow for T14 production independently of the mature AChE-R protein, providing a molecular basis for the observed correspondence [31]. This proposed mechanism could explain how T14 can be produced from AChE-R mRNA despite the absence of exon 6 in the mature protein and provides a molecular basis for the observed correlation between AChE-R expression and T14 levels during early development. Definitive resolution of these mechanisms will require advanced functional profiling, such as polysome profiling or ribosome footprinting, to confirm whether distinct translational initiation events occur during development. The demonstration that exogenous T30 selectively upregulates AChE-R in neuronal cell lines supports the existence of a feedforward mechanism in which T14 increases production of its own precursor. This positive feedback loop, physiological in the developing brain, may become a driver of neurodegeneration when inappropriately reactivated in the mature brain. These findings suggest that therapeutic strategies targeting this feedforward mechanism may offer a promising approach for intervention in Alzheimer’s disease by disrupting the cycle of AChE-R upregulation and T14 production. More broadly, further investigation of this pathway may help define a clinically actionable means of detecting early-stage pathology (i.e., through utilising T14 as a biomarker) and disrupting T14 signalling upstream of amyloid (i.e., through selective targeting of AChE isoforrms), potentially enabling intervention at an earlier stage of the disease process before downstream amyloid pathology is established. Furthermore, translating these findings will benefit from emerging bioanalytical platforms, such as nanozyme-integrated biosensors, which offer enhanced sensitivity for tracking microscale AChE dynamics in translational and point-of-care settings [37].

## Data Availability

The data that support the findings of this study are available from the corresponding author upon reasonable request.

## Supplementary Materials

The following supporting information can be downloaded at: https://www.mdpi.com/article/10.3390/biom16091336/s1, Figure S1: The original western blot images of Figure 2; Figure S2: The original western blot images of Figure 3.
