# Supplementary material for: A Novel Regulatory System in Brain Development and Death Driven by a Bioactive Peptide Linked to a Specific Isoform of Acetylcholinesterase
Source: Biomolecules. 2026 Sep 15;16(9):1336. doi: 10.3390/biom16091336 (PMC13604771; doi:10.3390/biom16091336)

Figure S1: The original western blot images of Figure 2

Figure 2A. Immunoneutralisation of T14

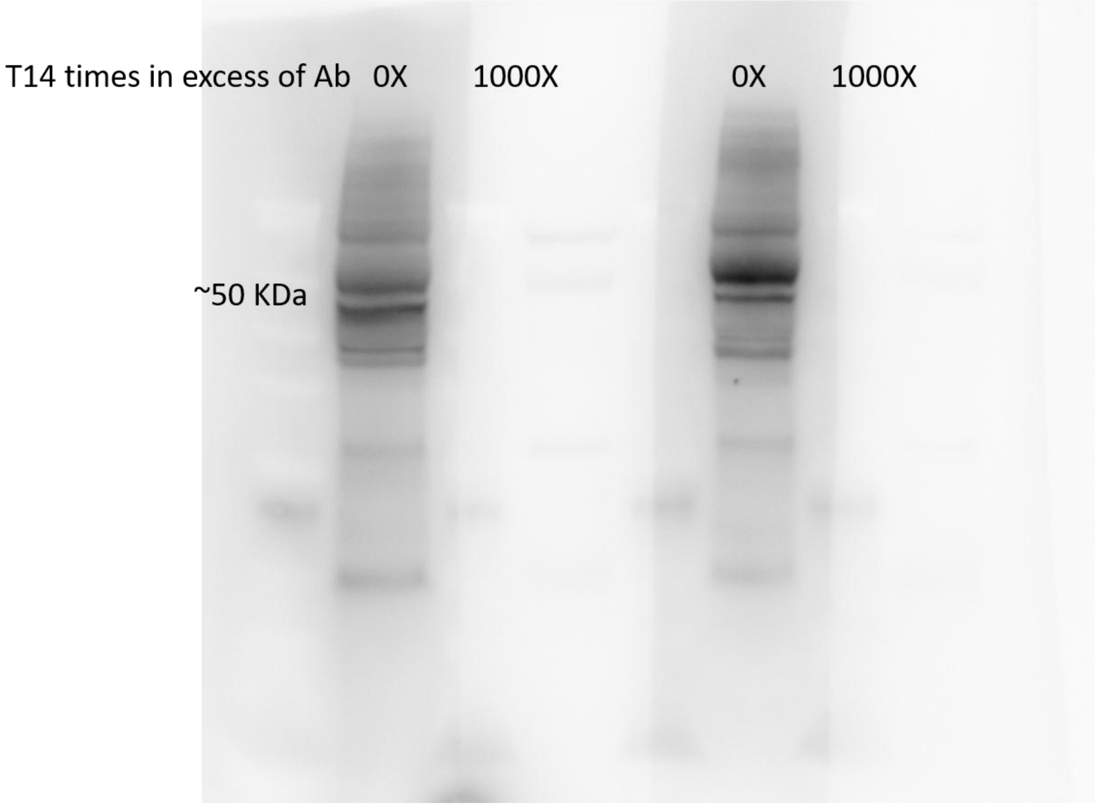

Figure 2A/B/C/D. Cortical T14.

Gel 1.

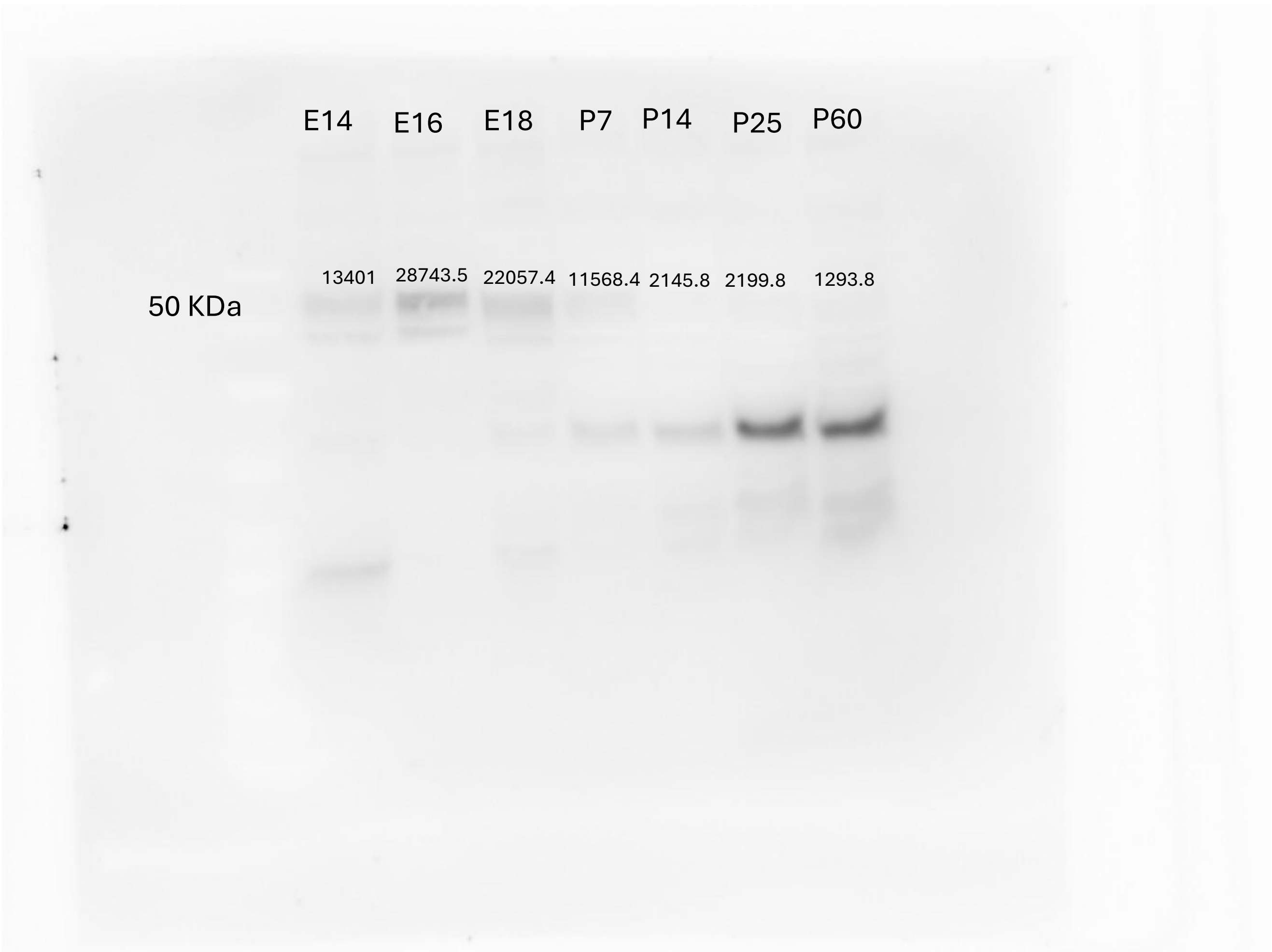

Figure 2A/B/C/D. Cortical T14.

Gel 2.

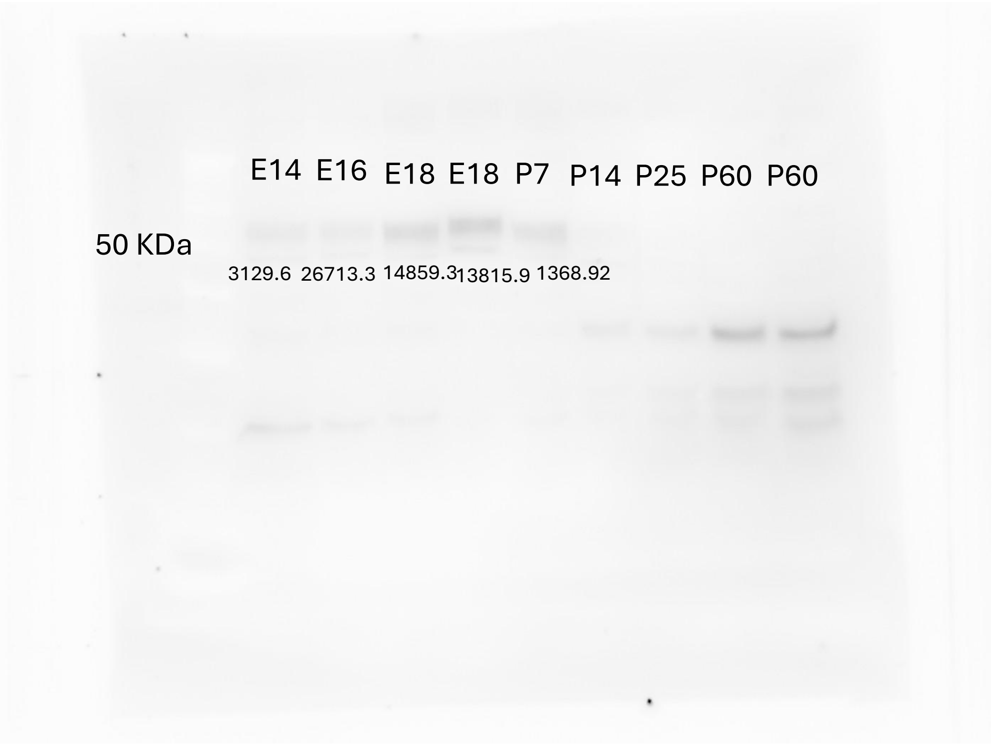

Figure 2A/B/C/D. Cortical T14.

Gel 3.

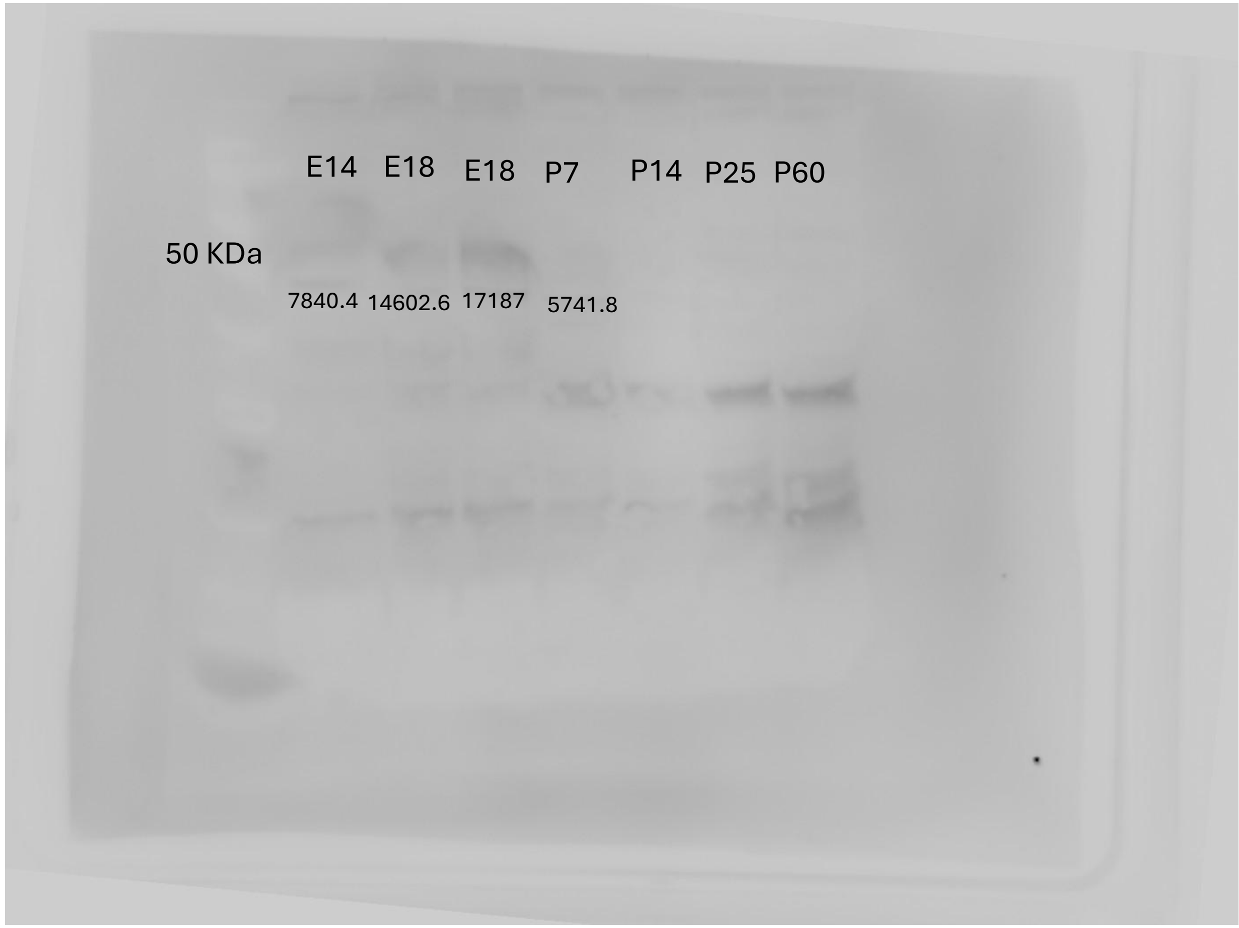

Figure 2A/B/C/D. Cortical T14.

Gel 4.

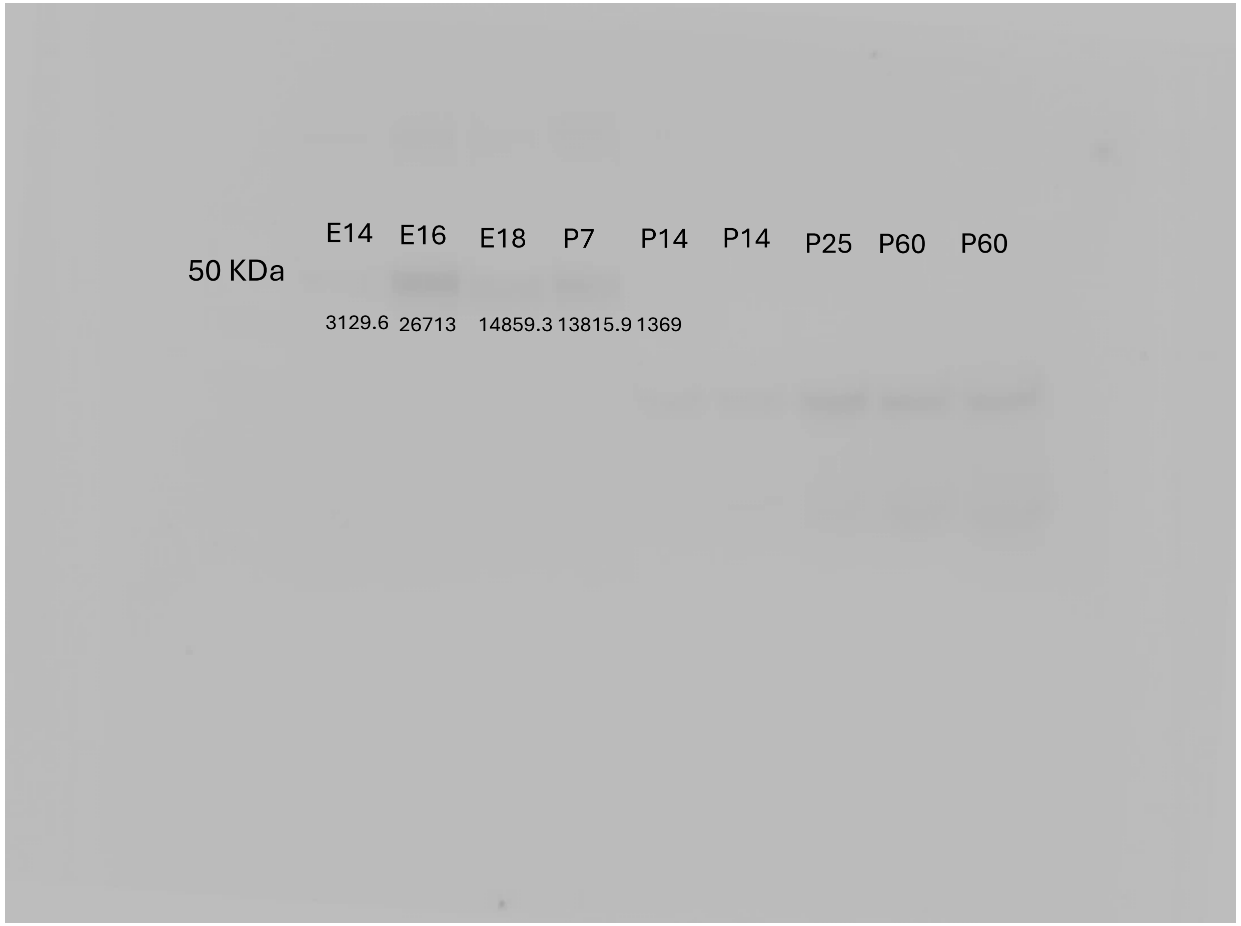

Figure 2A/B/C/D. Cortical T14.

Gel 5.

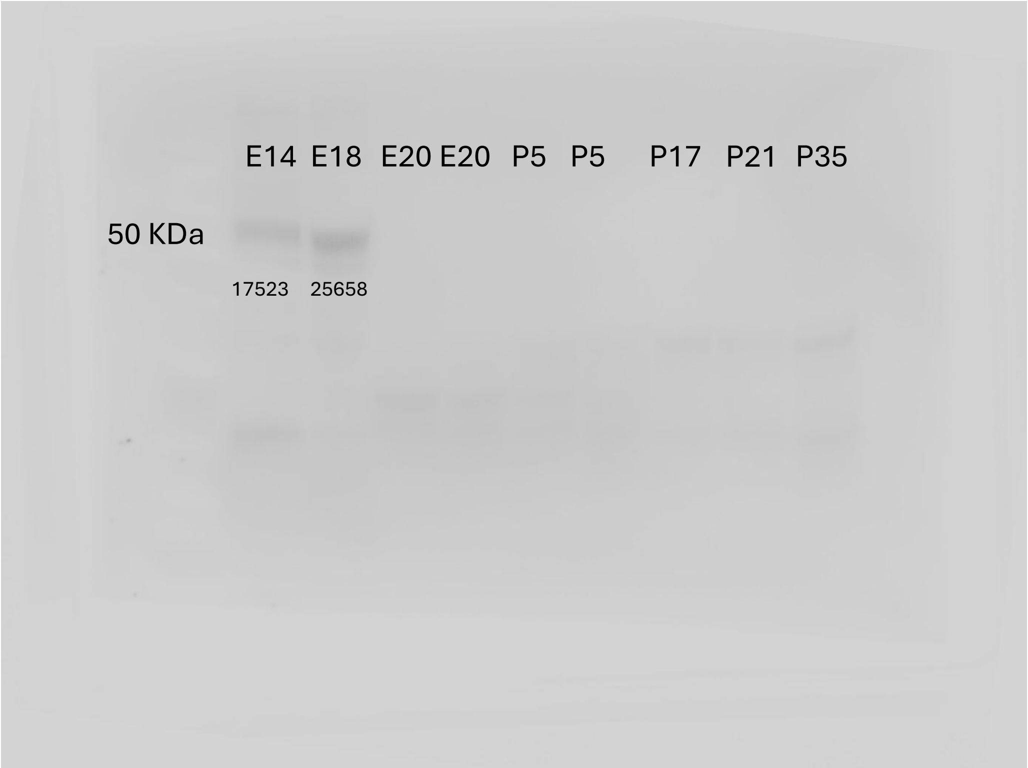

Figure S2: The original western blot images of Figure 3

Figure 3A. Subcortical T14. Gel 1

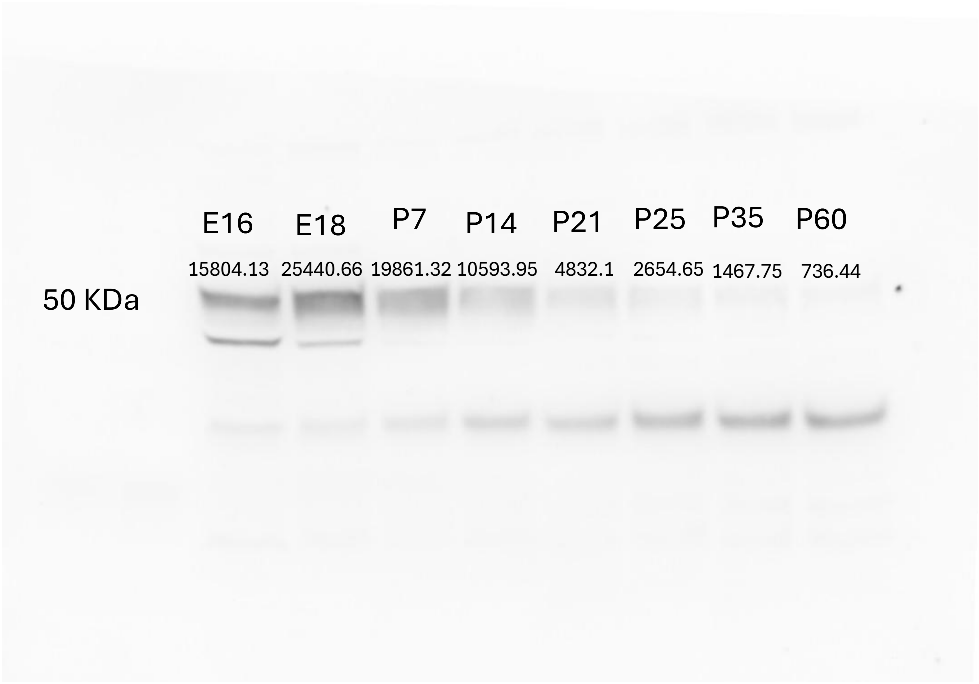

Figure 3A. Subcortical T14.

Gel 2

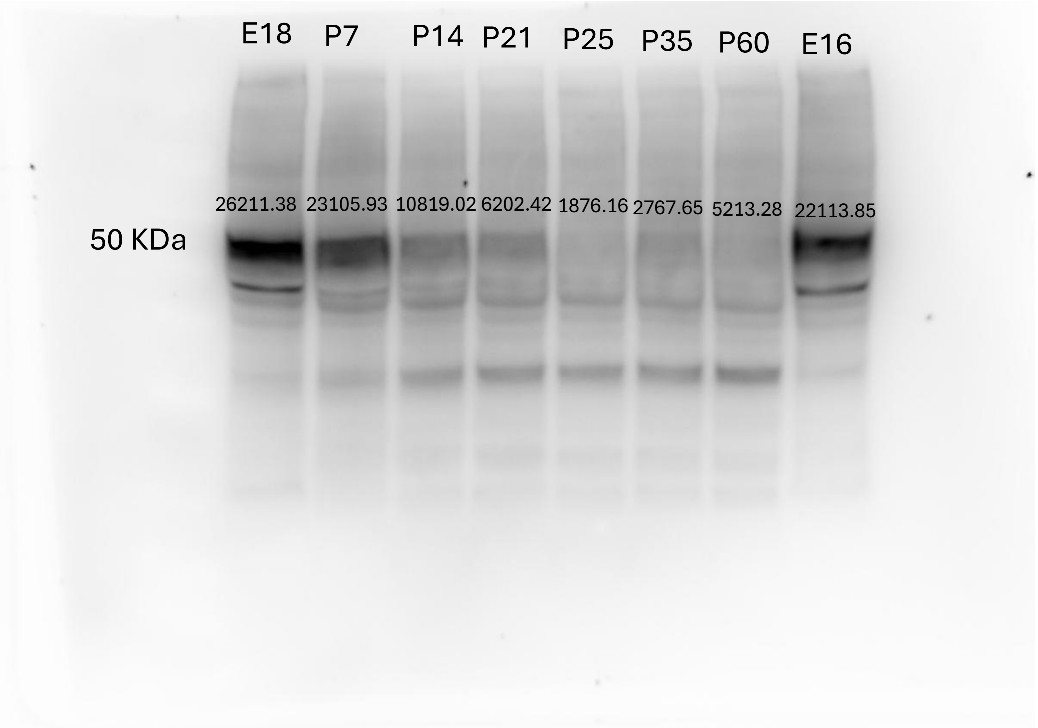

Figure 3A. Subcortical T14.

Gel 3

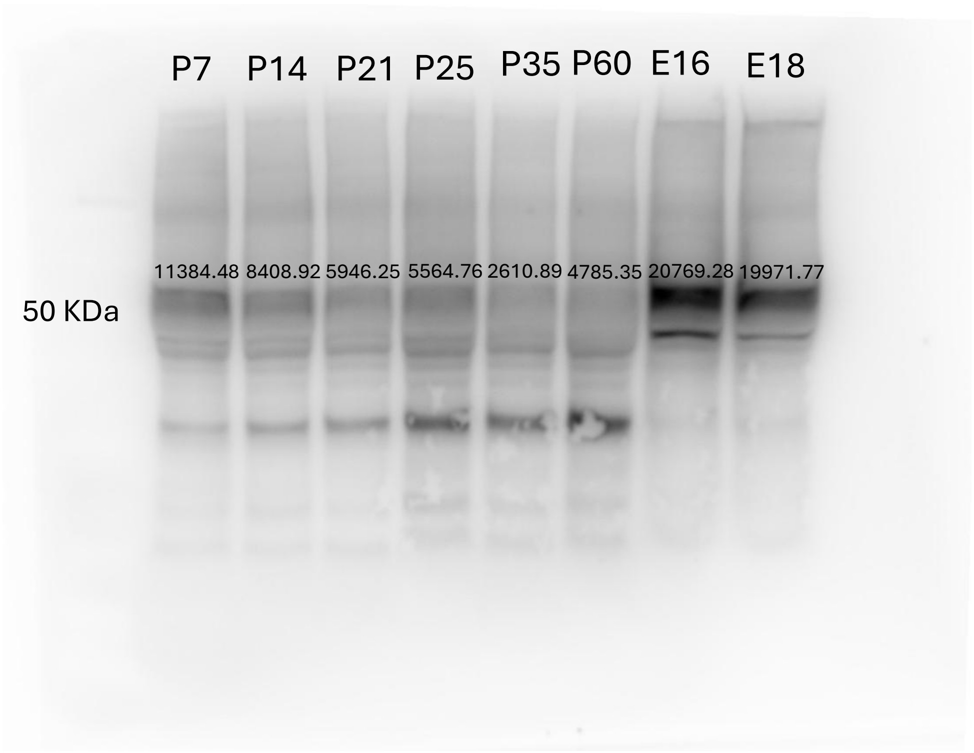

Figure 3A. Subcortical T14.

Gel 4

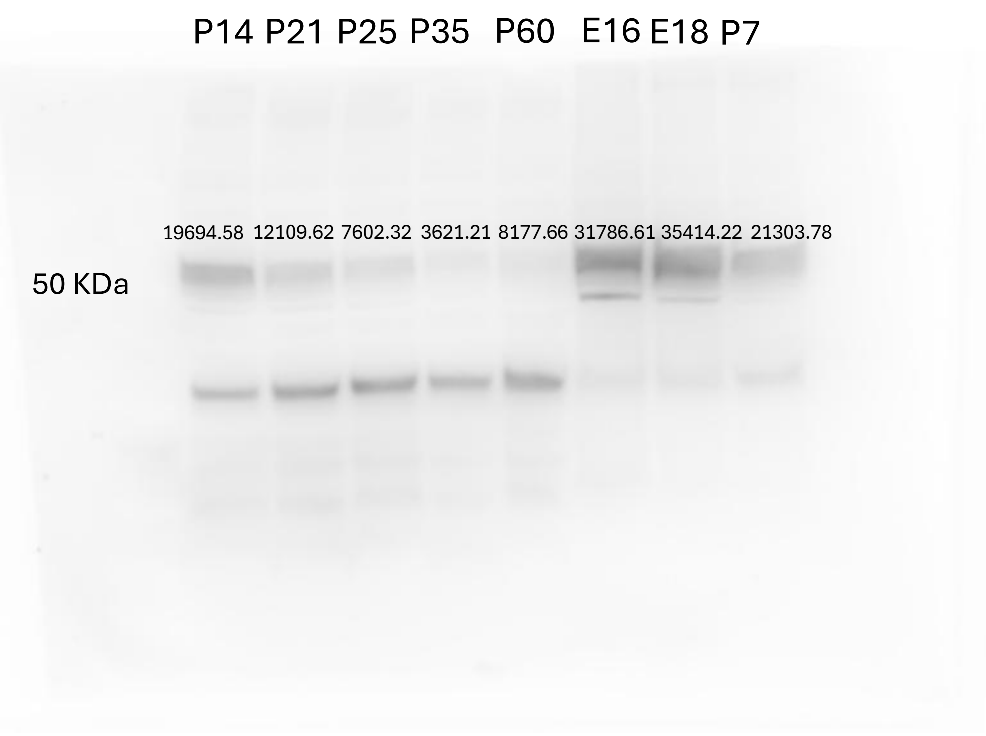

Figure 3A. Subcortical T14.

Gel 5

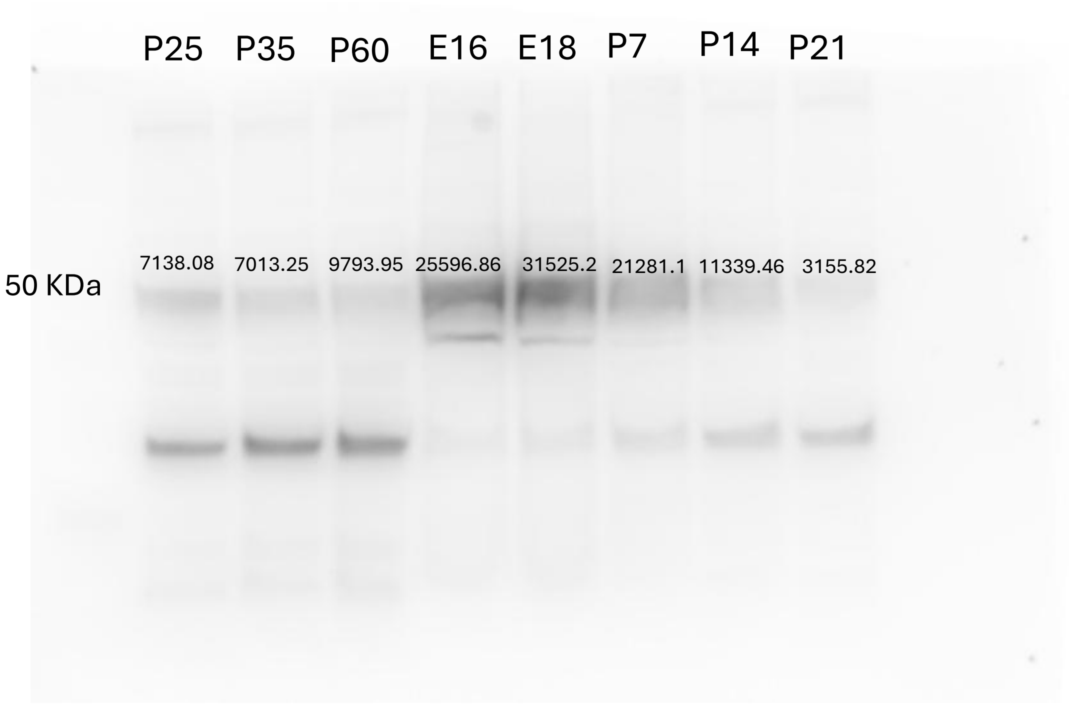

Figure 3A. Subcortical T14.

Gel 6

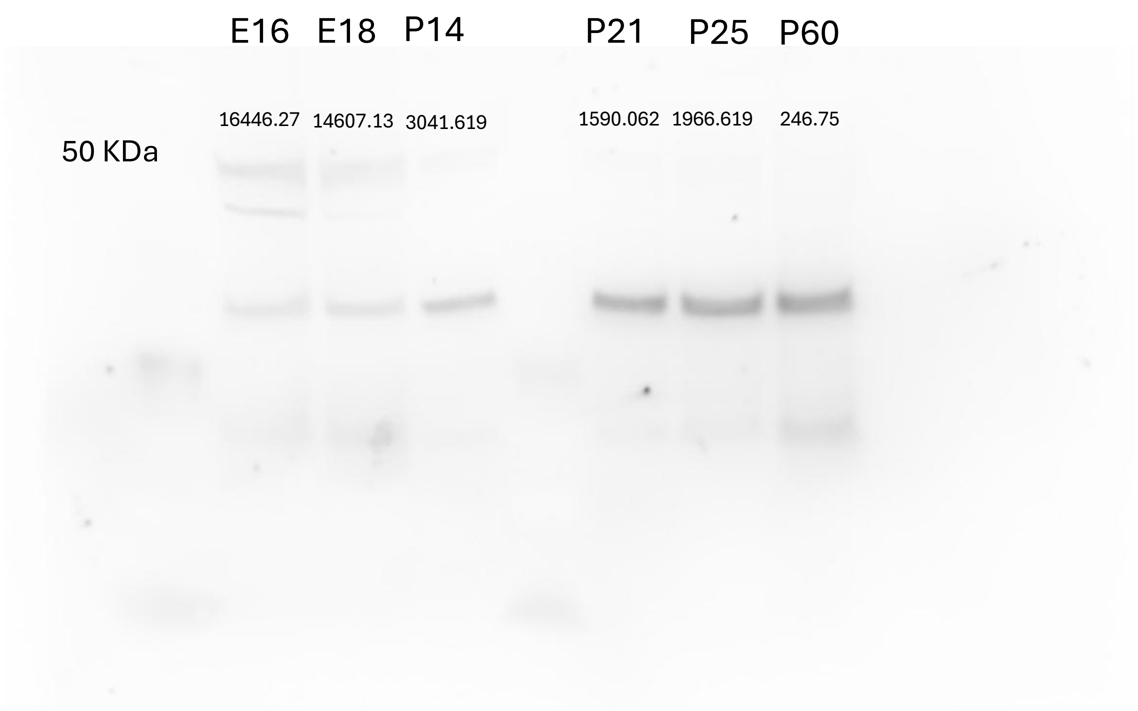

Figure 3A. Subcortical T14.

Gel 7

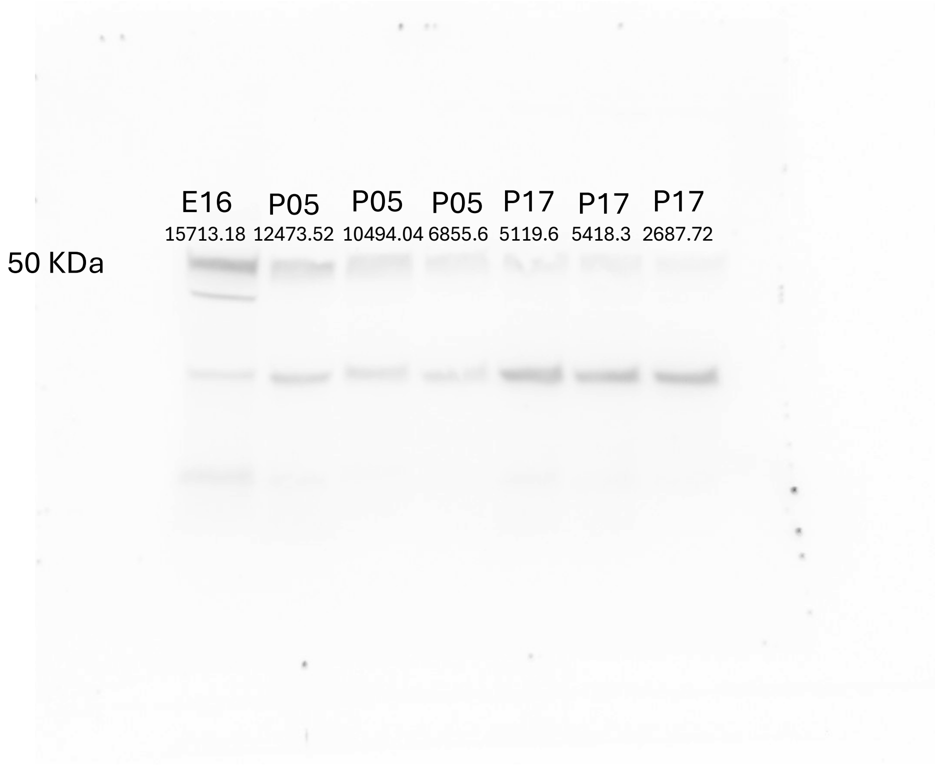

Figure 3B. Subcortical NeuN.

Gel 1.

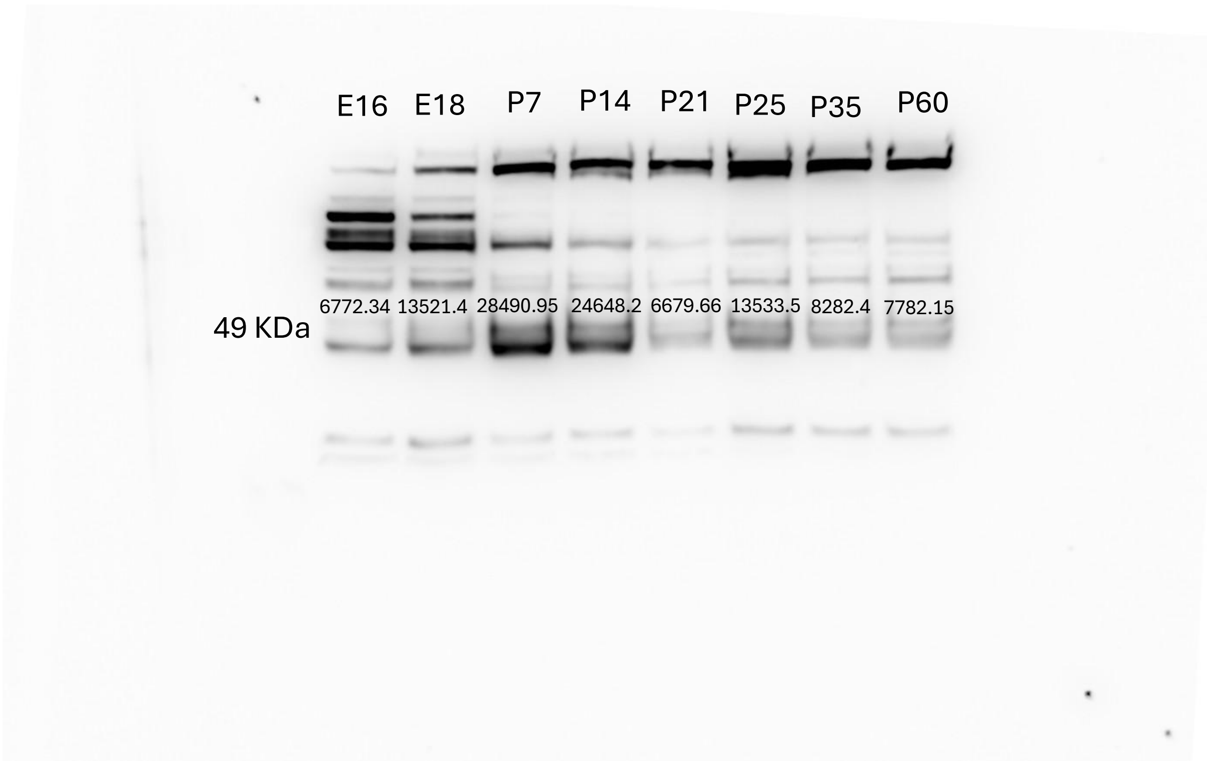

Figure 3B. Subcortical NeuN.

Gel 2.

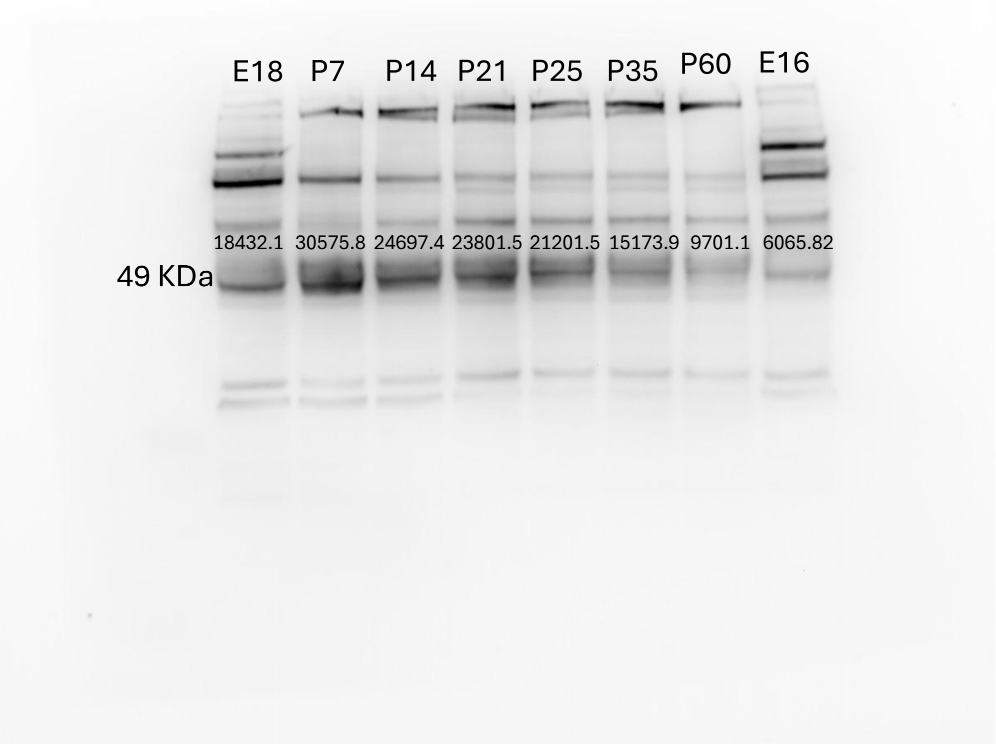

Figure 3B. Subcortical NeuN.

Gel 3.

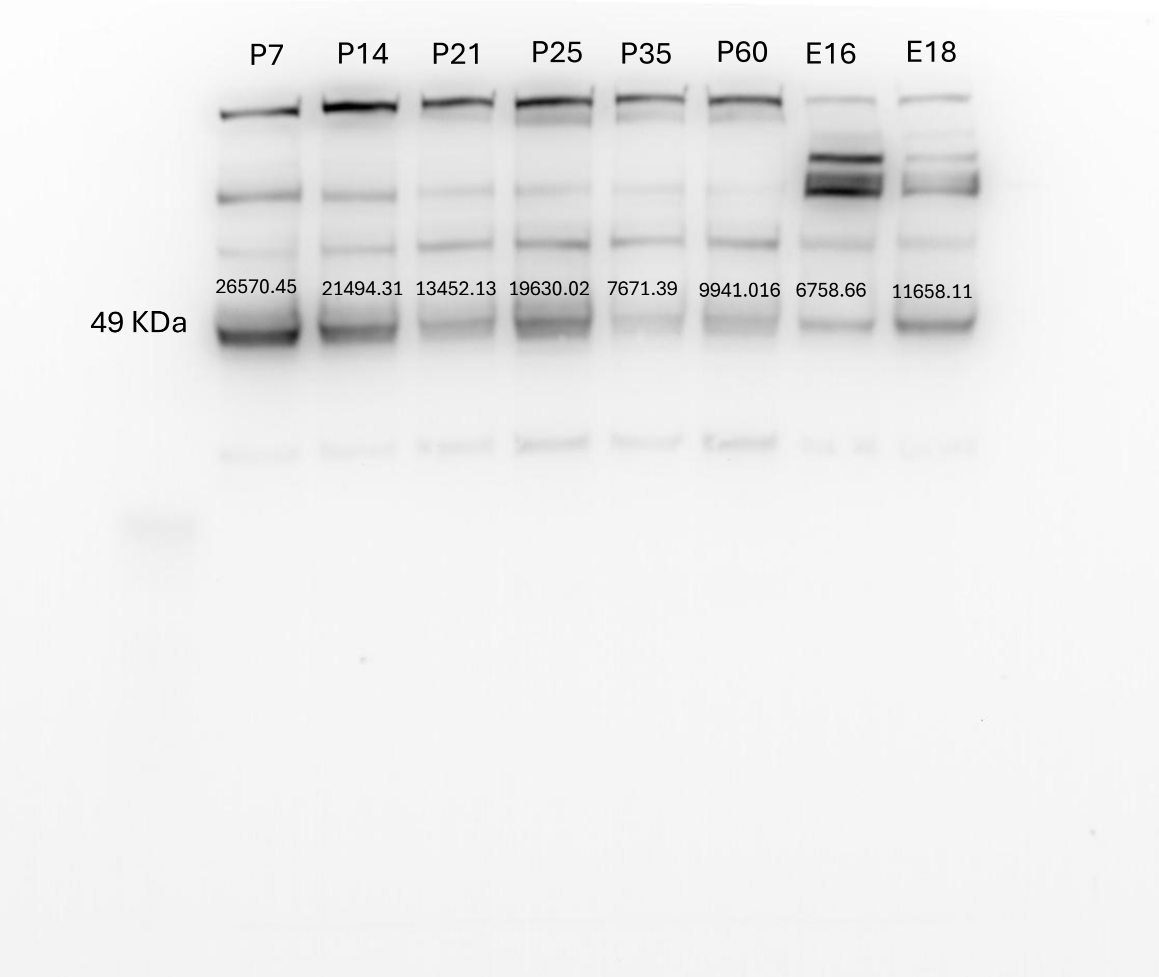

Figure 3B. Subcortical NeuN.

Gel 4.

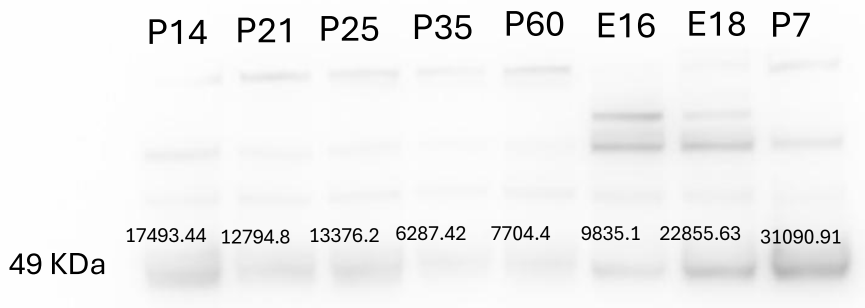

Figure 3B. Subcortical NeuN.

Gel 5.

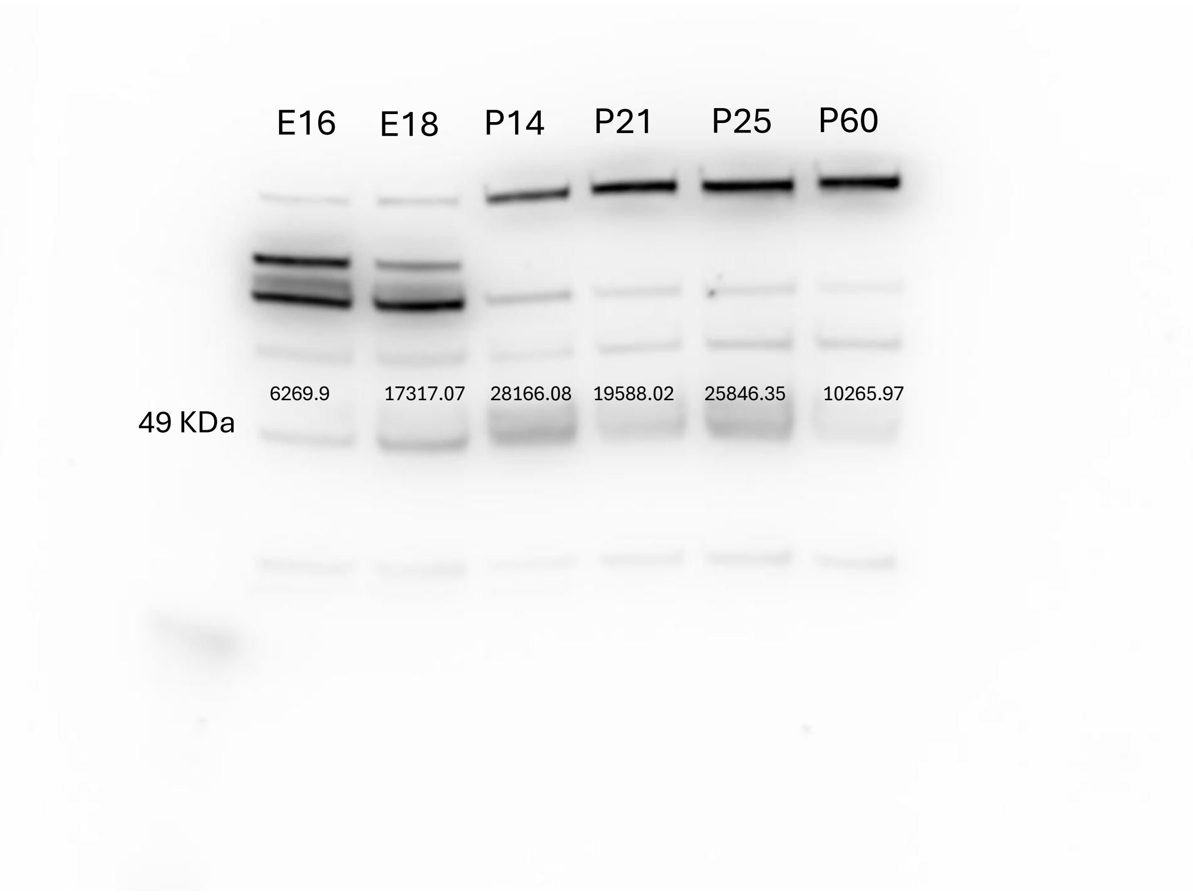

Figure 3C. T14 whole mouse brain.

Gel 1. P7 (M= mouse)

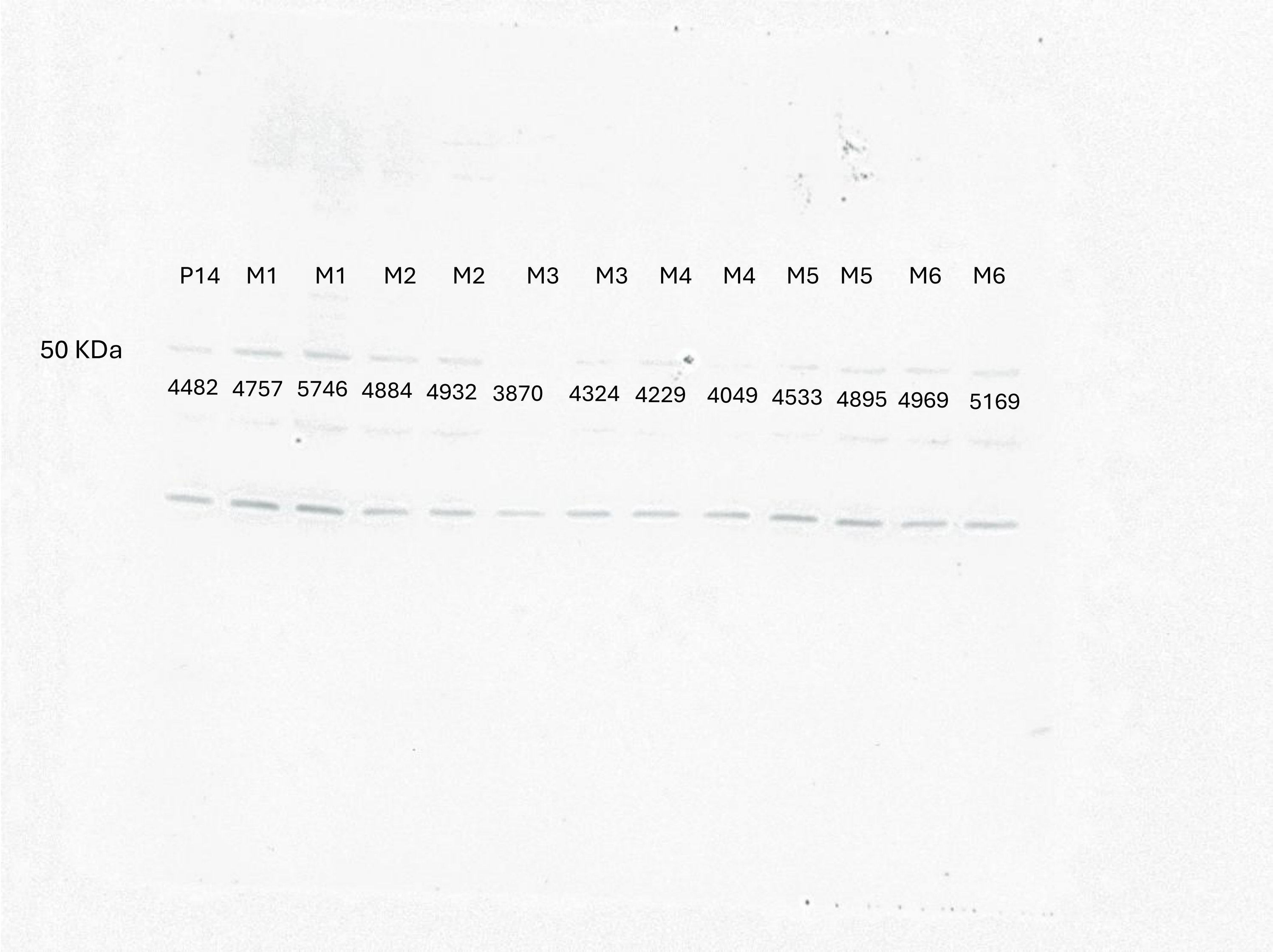

Figure 3C. T14 whole mouse brain.

Gel 2. P14

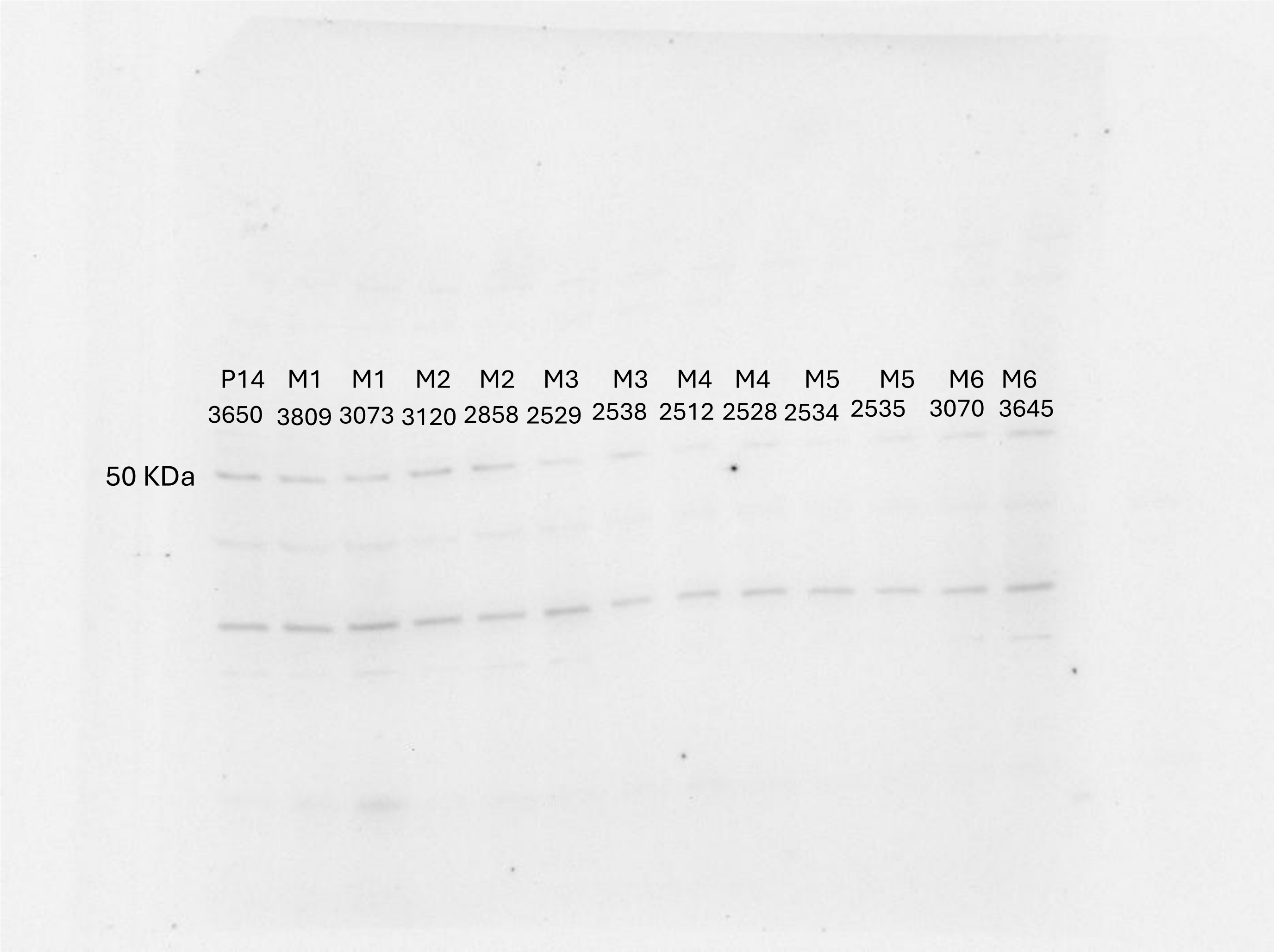

Figure 3C. T14 whole mouse brain.

Gel 3. P21

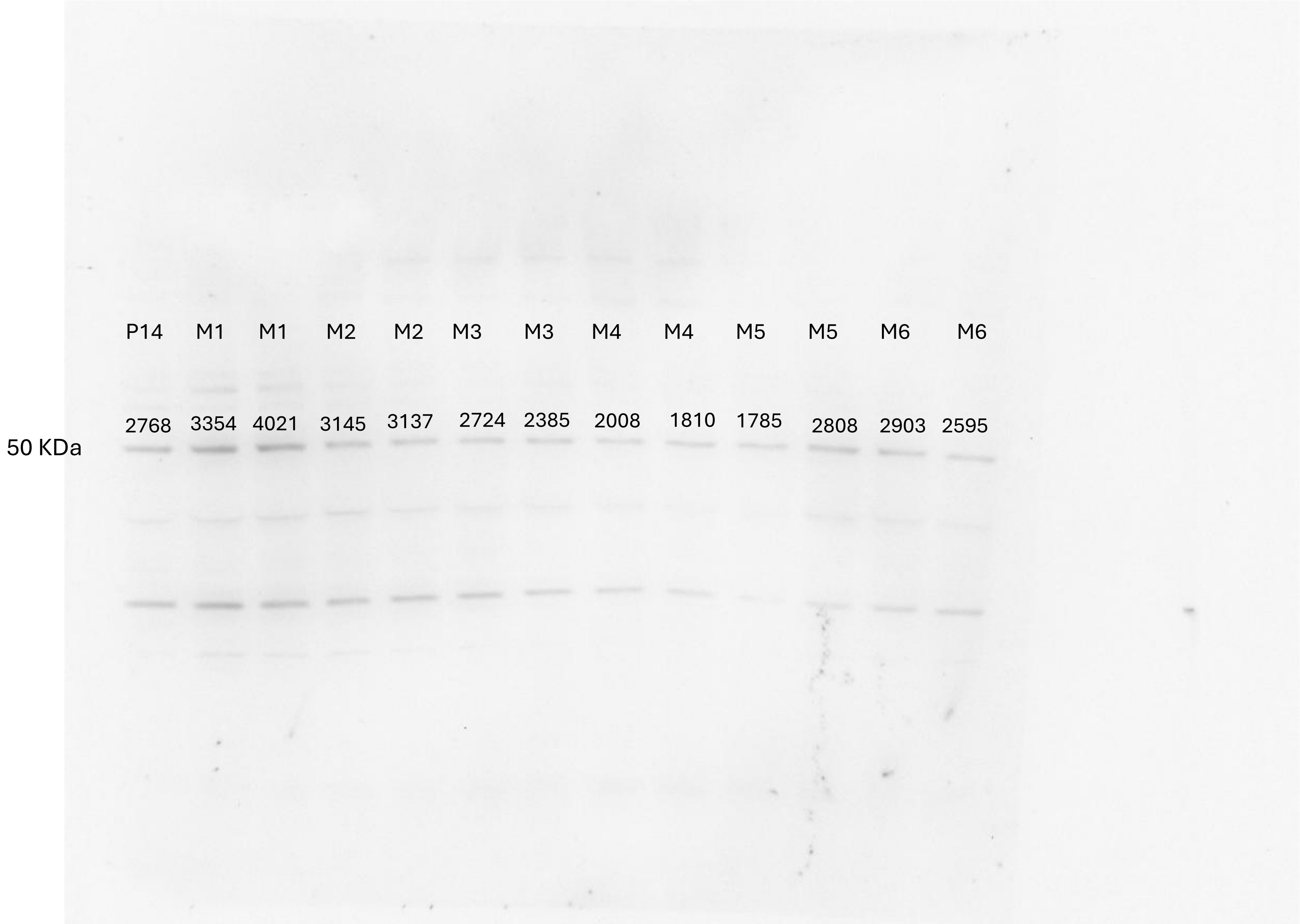

Figure 3C. T14 whole mouse brain.

Gel 4. P60

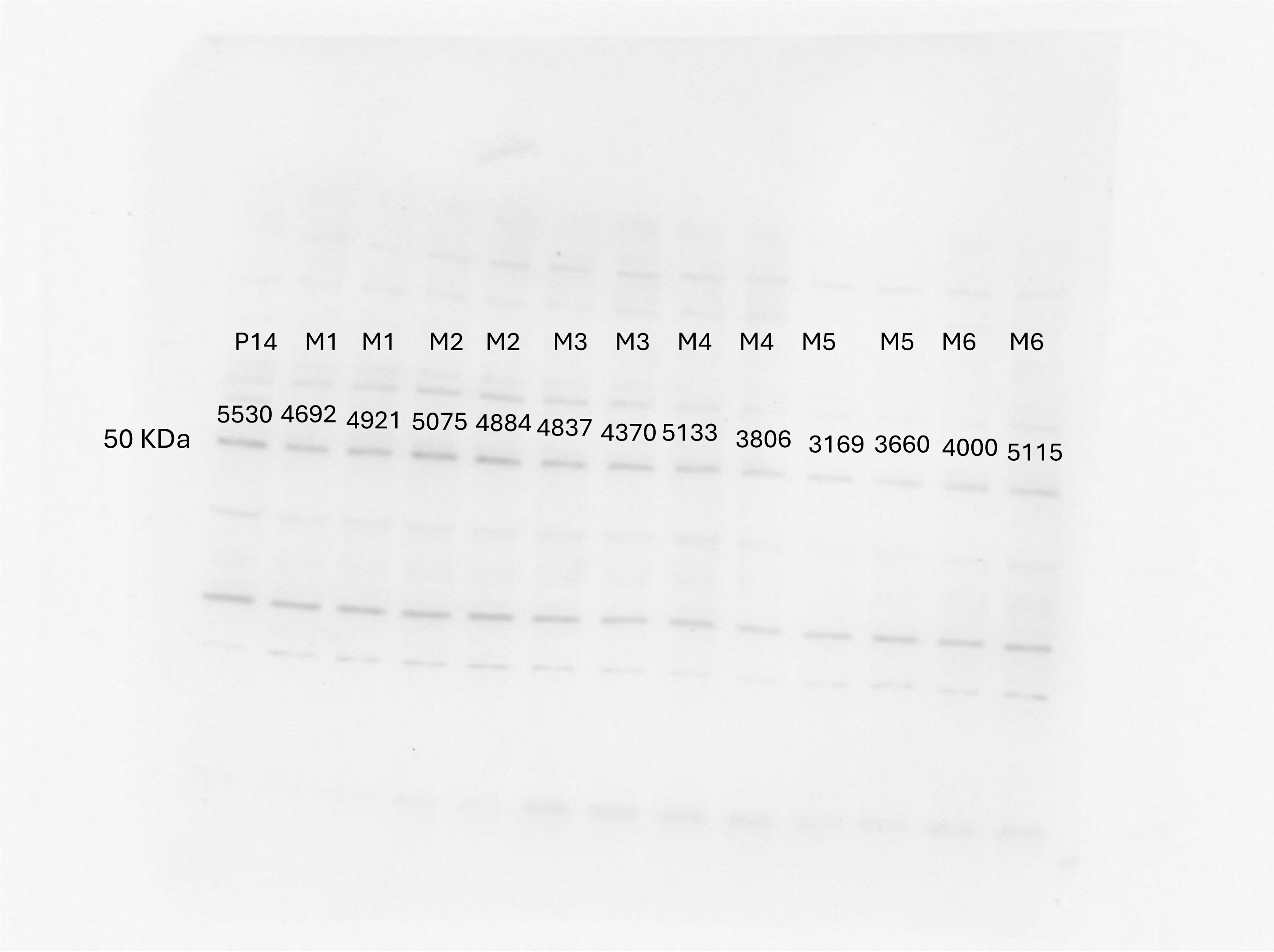

Figure 3D. pTau Whole mouse brain.
Gel 1. P7

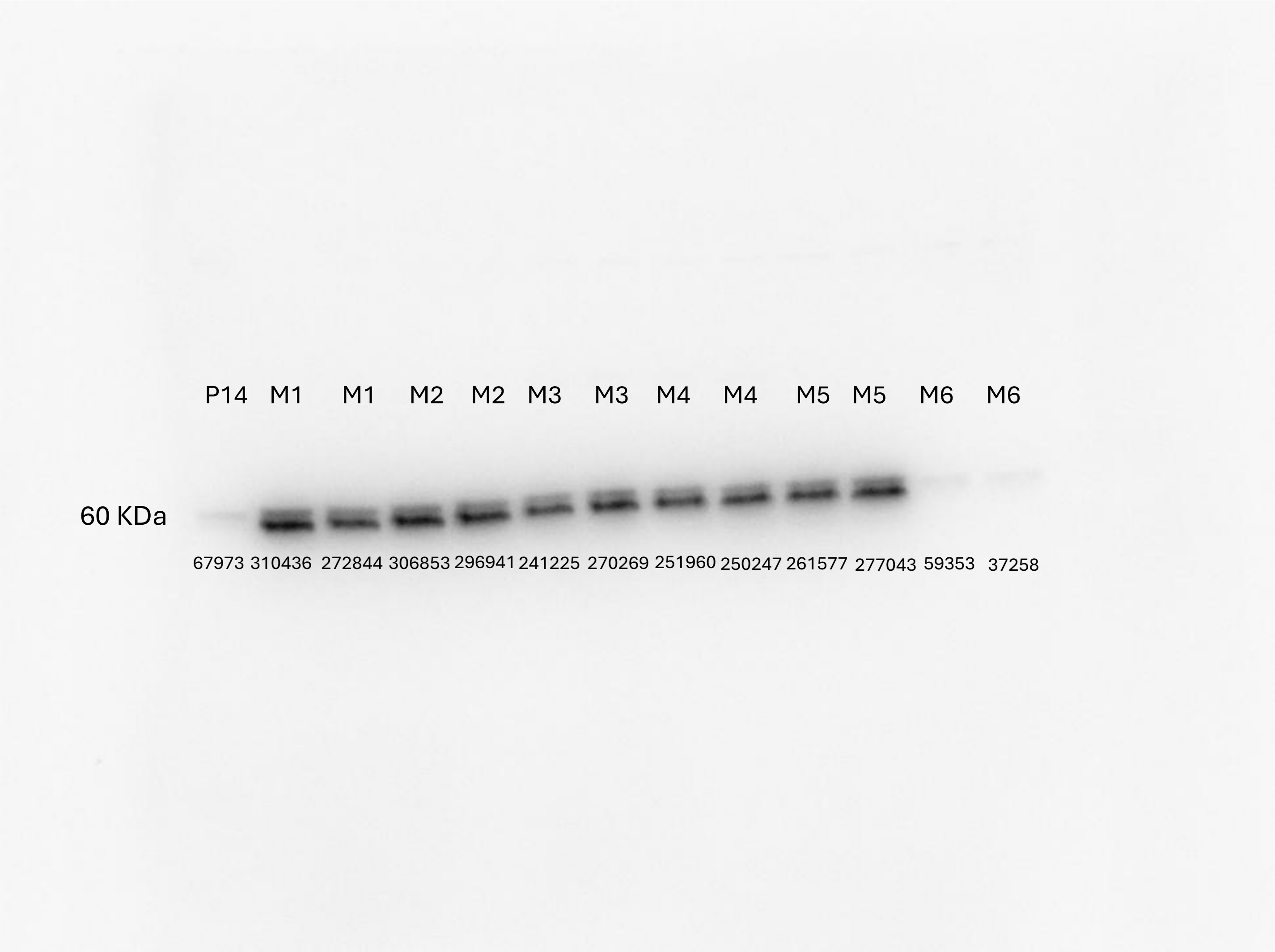

Figure 3D. pTau Whole mouse brain.

Gel 2. P14

M1 M1 M2 M2 M3 M3 M4 M4 M5 M5 M6 M6

60 KDa

222081 229791 185828 187654 219568 228728 172515 183552 184808 182175 131060 128568

Figure 3D. pTau Whole mouse brain.

Gel 3. P21

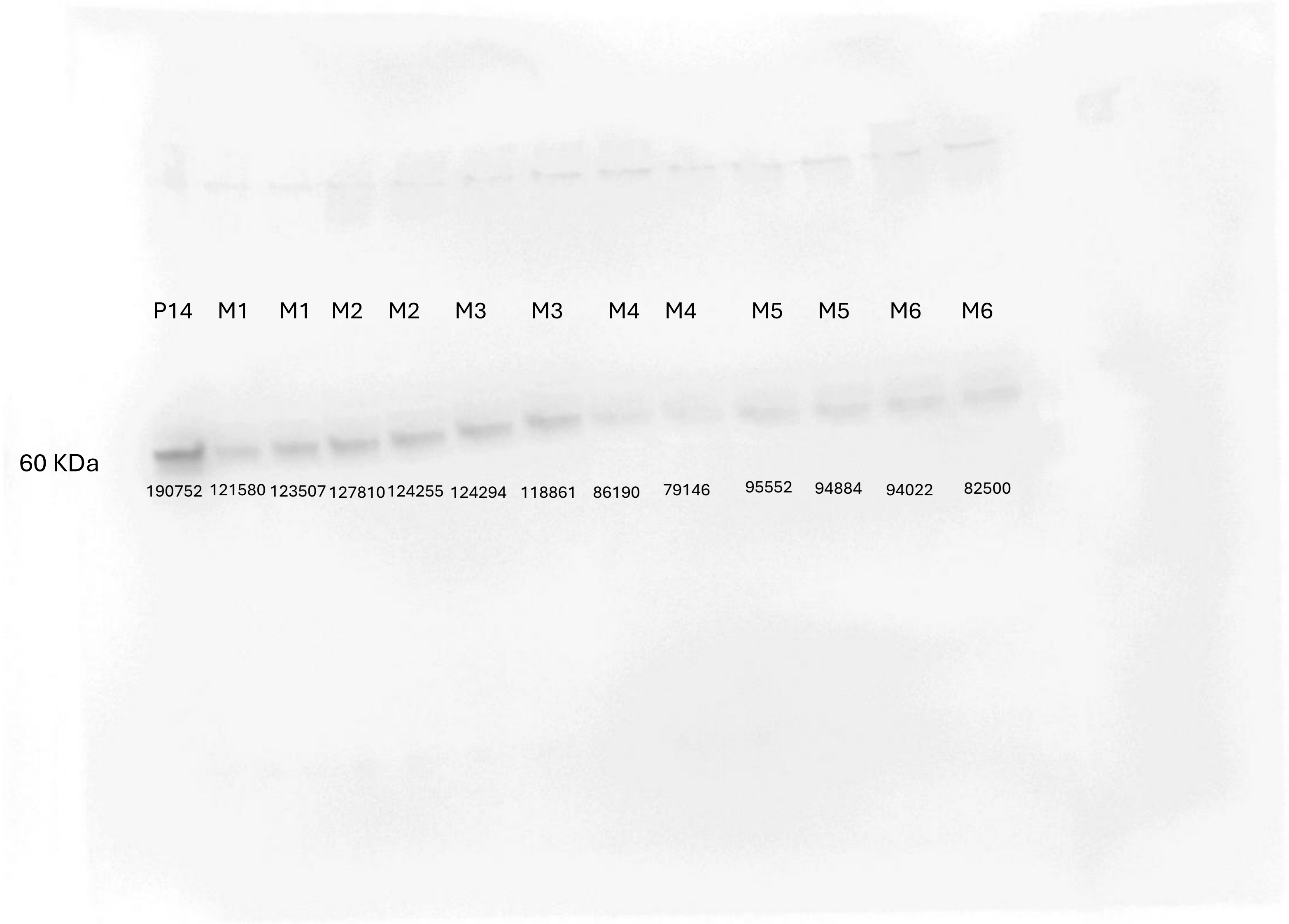

Figure 3D. pTau Whole mouse brain.

Gel 4. P60

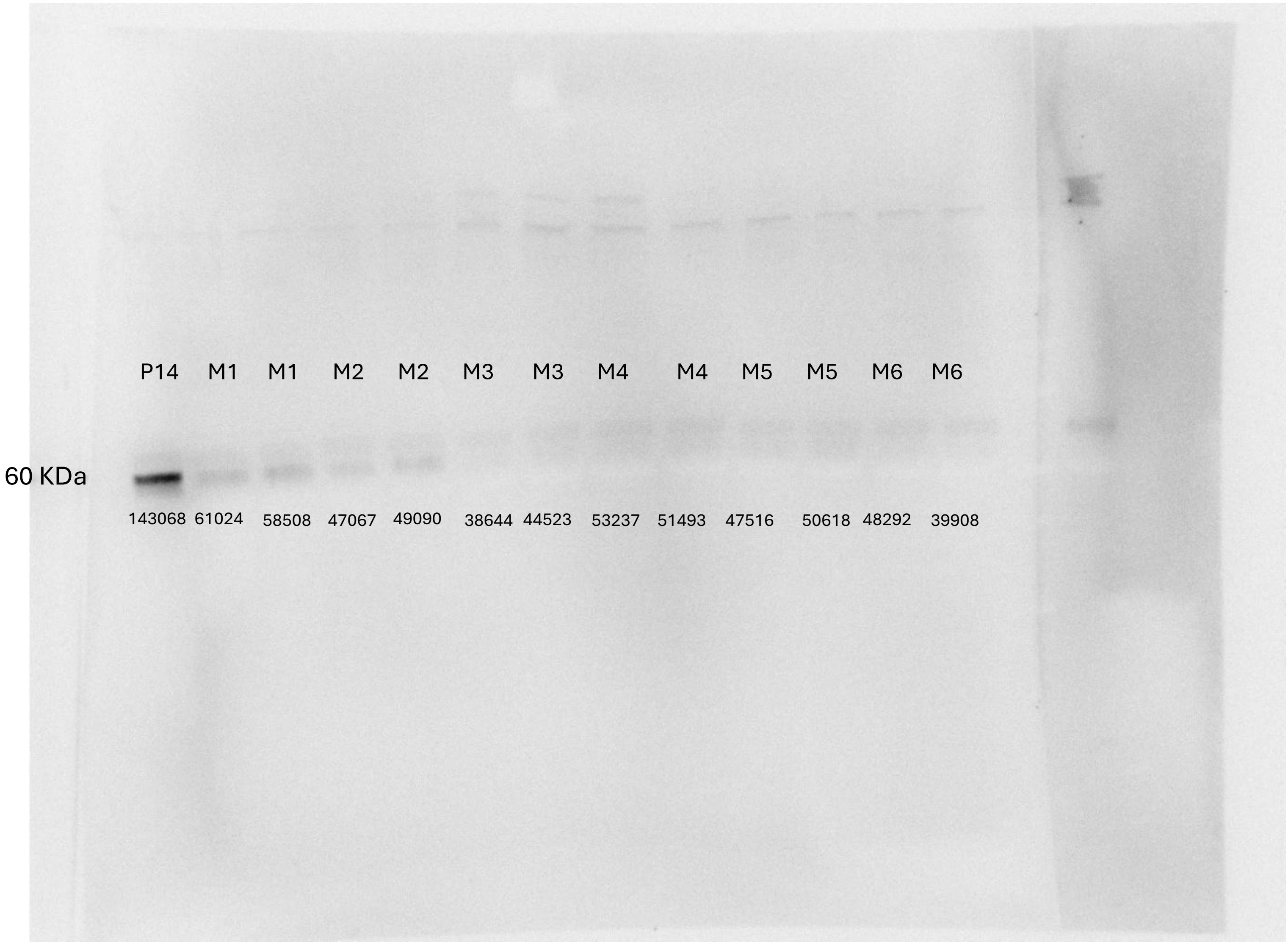

Supplement: Supplementary file 1 [file biomolecules-16-01336-s001.zip › biomolecules-4528848-supplementary.pdf]
